# Integrating genomic and spatial analyses to describe tuberculosis transmission: a scoping review

**DOI:** 10.1016/j.lanmic.2025.101094

**Published:** 2025-04-11

**Authors:** Yu Lan, Isabel Rancu, Melanie H Chitwood, Benjamin Sobkowiak, Kate Nyhan, Hsien-Ho Lin, Chieh-Yin Wu, Barun Mathema, Tyler S Brown, Caroline Colijn, Joshua L Warren, Ted Cohen

**Affiliations:** Department of Epidemiology of Microbial Diseases, Yale School of Public Health, New Haven, CT, USA; Department of Epidemiology of Microbial Diseases, Yale School of Public Health, New Haven, CT, USA; Department of Epidemiology of Microbial Diseases, Yale School of Public Health, New Haven, CT, USA; Department of Epidemiology of Microbial Diseases, Yale School of Public Health, New Haven, CT, USA; Department of Environmental Health Sciences, Yale School of Public Health, New Haven, CT, USA; Harvey Cushing/John Hay Whitney Medical Library, Yale University, New Haven, CT, USA; Institute of Epidemiology and Preventive Medicine, National Taiwan University College of Public Health, Taipei, Taiwan; Institute of Epidemiology and Preventive Medicine, National Taiwan University College of Public Health, Taipei, Taiwan; Mailman School of Public Health, Columbia University, New York City, NY, USA; Section of Infectious Diseases, Boston University Chobanian & Avedisian School of Medicine, Boston, MA, USA; Department of Mathematics, Simon Fraser University, Burnaby, BC, Canada; Department of Biostatistics, Yale School of Public Health, New Haven, CT, USA; Department of Epidemiology of Microbial Diseases, Yale School of Public Health, New Haven, CT, USA

## Abstract

Tuberculosis remains a leading cause of infection-related mortality, and efforts to reduce its incidence have been hindered by an incomplete understanding of local *Mycobacterium tuberculosis* transmission dynamics. Advances in pathogen sequencing and spatial analysis have created new opportunities to map *M tuberculosis* transmission patterns more precisely. In this scoping review, we searched for studies combining pathogen genetics and location data to analyse the spatial patterns of *M tuberculosis* transmission and identified 142 studies published between 1994 and 2024. Secular changes in genetic methods were observed, with genome sequencing approaches largely replacing lower-resolution genotyping methods since 2020. The included studies addressed four primary research questions: how are tuberculosis cases and *M tuberculosis* transmission clusters geographically distributed; do spatially concentrated *M tuberculosis* clusters exist, and where are these areas located; when spatial concentration occurs, what host, pathogen, or environmental factors contribute to these patterns; and do identifiable relationships exist between the spatial proximity of tuberculosis cases and the genetic similarity of the *M tuberculosis* isolates infecting these individuals? Collectively, in this Review, we examined the available study data, evaluated the analytical requirements for addressing these questions, and discussed opportunities and challenges for future research. We found that the integration of spatial and genomic data can inform a detailed understanding of local *M tuberculosis* transmission patterns, but improved study designs and new analytical methods to address gaps in sampling completeness and to integrate additional movement data are needed to fully realise the potential of these tools.

## Editorial note

The Lancet Group takes a neutral position with respect to territorial claims in published maps.

## Introduction

Tuberculosis remains a leading infectious cause of mortality, and global efforts to reduce its incidence have faced persistent challenges.^1^ Tuberculosis is caused by the respiratory transmission of *Mycobacterium tuberculosis*, and approaches that can help to identify local transmission patterns are required for guiding targeted interventions to reduce incidence in endemic settings.^2^ Rapidly increasing accessibility, resolution, and affordability of pathogen sequencing and the development of methods for integrating spatial and genetic data have created new opportunities for describing local pathogen transmission dynamics.^3–7^

In 2018, Shaweno and colleagues published a review of studies on the spatial epidemiology of tuberculosis and identified 25 studies that combined spatial and genotyping methods.^8^ At that time, the authors did not identify studies that used genomic sequencing techniques (eg, whole-genome sequencing [WGS]) in combination with spatial analysis. WGS typically examines over 90% of the *M tuberculosis* genome, whereas genotyping methods capture 1% or less.^9,10^ With advancements in pathogen genetics and spatial data analysis, several studies have used these technologies to investigate *M tuberculosis* transmission in communities.^11^

Given these developments and the diversity of methodologies, we present a scoping review of studies that combine genomic and spatial analyses to identify local patterns of *M tuberculosis* transmission. We report the types of genetic and spatial data included and categorise them based on the primary research questions addressed. We provide a summary of statistical methods used to analyse different types of spatial and genomic data to describe *M tuberculosis* transmission and provide a mapping of these methods to the types of data examined and the research questions posed. Based on these findings, we evaluate challenges and opportunities for future studies that integrate WGS and spatial analysis to advance the understanding of *M tuberculosis* transmission.

## Methods

### Search strategy and selection criteria

Using our search strategy, we identified peer-reviewed studies in English that used both genetic and spatial data to describe *M tuberculosis* transmission patterns. We searched for papers published before June 3, 2024, in four databases: PubMed, Web of Science Core Collection, Embase (Ovid), and Scopus. Our search included three groups of terms: genomic terms (eg, “genom*”), spatial terms (eg, “geograph*”), and tuberculosis-related terms (eg, “tuberculosis”). A detailed search strategy for this Review, including search terms and relevant information, is provided in the appendix (p 1) and in the supplement of our published protocol on the Open Science Framework registries.^12^ We used the reference management software Endnote and Covidence for screening and extraction of the included studies. The artificial intelligence tools implemented in Covidence were not used.

### Inclusion and exclusion criteria

Studies focusing on questions related to the transmission of *M tuberculosis* between humans and incorporating both genetic and spatial data were included in the Review. Genetic data could include those obtained from genotyping methods, such as spacer oligonucleotide typing (spoligotyping), variable-number tandem repeat of mycobacterial interspersed repetitive unit analysis (MIRU-VNTR), IS*C110* RFLP analysis, or genomic sequencing (ie, WGS; detailed in the appendix p 3). Spatial data could include point or areal locations. Studies that focused primarily on microbial evolution, including the long-range dispersal of *M tuberculosis* associated with human migration, were excluded from our Review. No authors from included studies were contacted. We did not include grey literature and we did not assess the quality of studies in this scoping review.

Following the removal of duplicate records, two reviewers (YL and IR) independently conducted a preliminary screening based on titles and abstracts, followed by full-text screening of studies meeting the inclusion criteria. Disagreements in eligibility assessments were resolved through discussion. The studies ultimately considered for inclusion were subsequently reviewed by the senior author (TC).

### Data extraction and analysis

Two reviewers (YL and IR) independently extracted information from full-text versions of the included studies using a standardised template (appendix p 2) and entered in a spreadsheet. The retrieved and charted data included the study setting, study design and duration, type of *M tuberculosis* genetic data, type of spatial data available, and analytical approaches used to integrate these genetic and spatial data. Discrepancies in extracted data were resolved through discussion, with adjudication by the senior author (TC). Our scoping review has been prepared according to the PRISMA-ScR guidelines,^13^ and the checklist is provided in the appendix (pp 25–27).

## Results

### Study selection and characteristics

**Figure 1:**
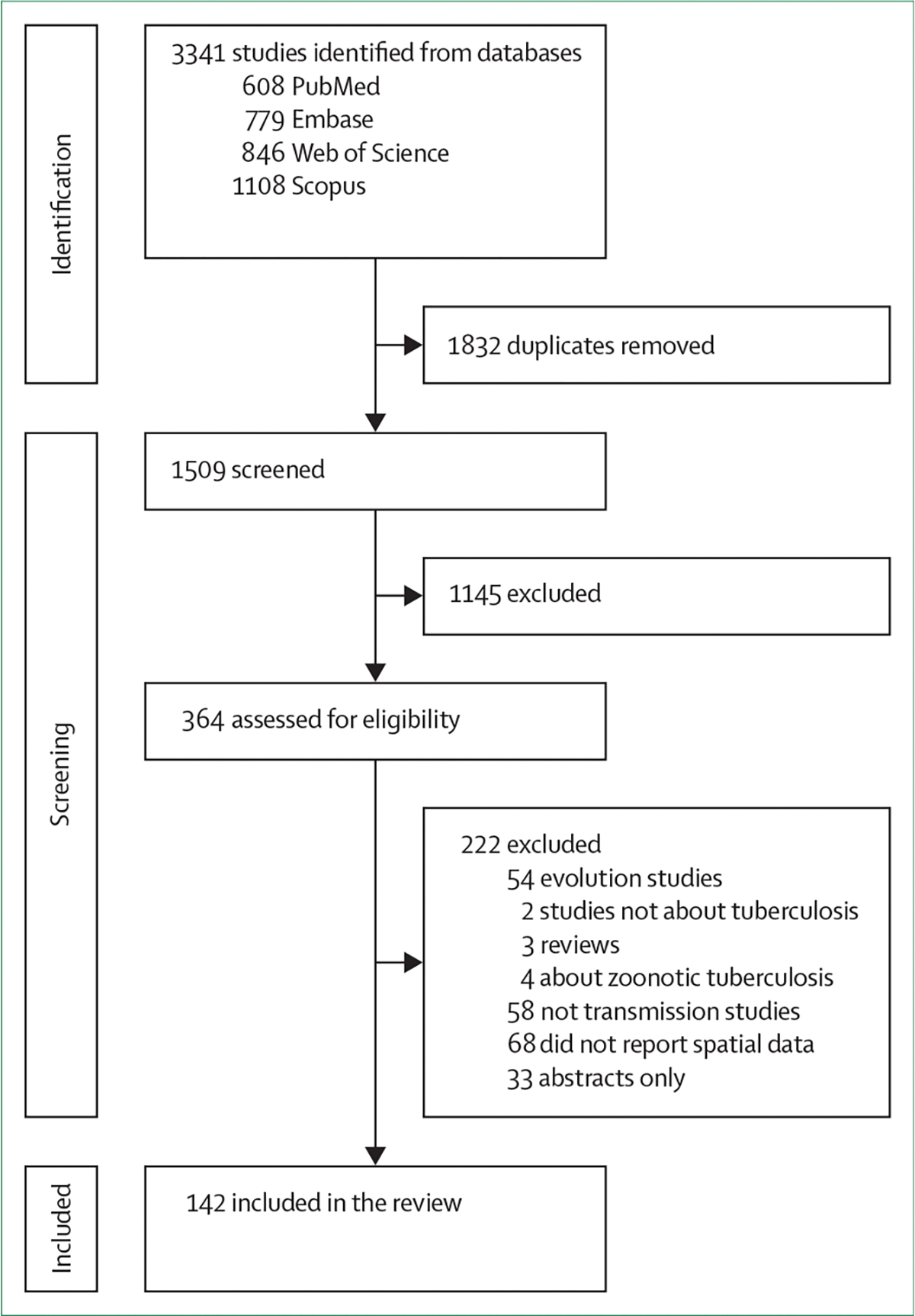
Flowchart of the search strategy and selection criteria

We retrieved 3341 studies from PubMed (n=608), Embase (n=779), Web of Science (n=846), and Scopus (n=1108). After the removal of duplicate studies (n=1832), the preliminary screening of titles and abstracts (n=1509) resulted in 364 articles selected for full-text screening. Based on the full-text review, 142 studies met the inclusion criteria (figure 1). A list of all included studies and abstracted data are provided in the appendix (pp 3–9).

### Study setting and design

**Figure 2:**
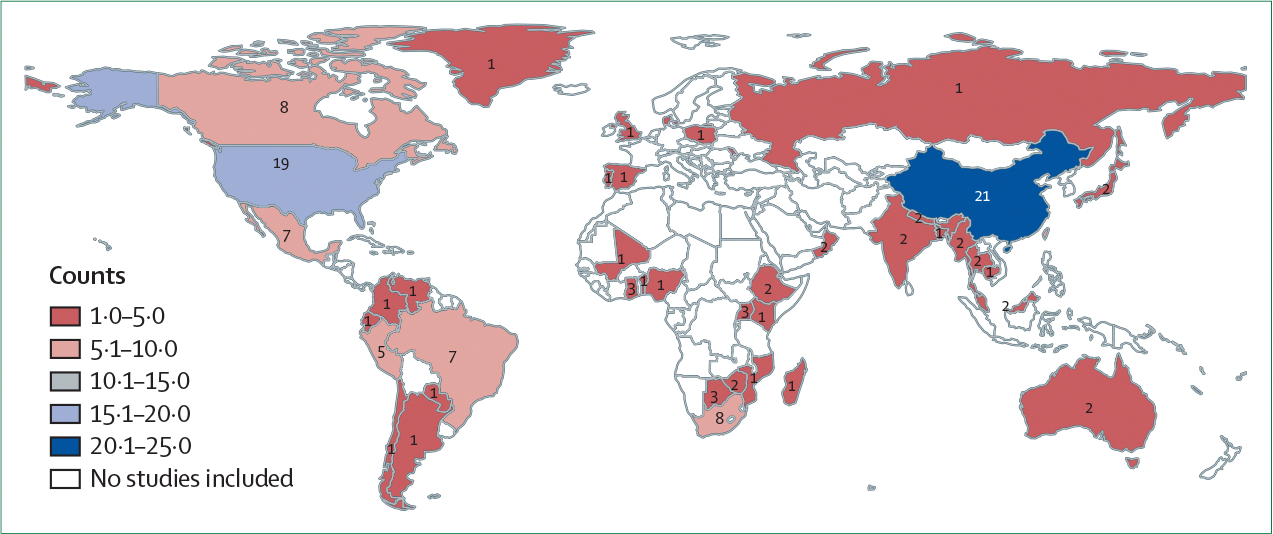
Country-wise distribution of the included studies

The 142 studies included in this Review were published between December, 1994, and May, 2024. Most studies (n=139) focused on *M tuberculosis* transmission within a single country or subnational setting, with the highest number of studies based in China (n=21), followed by the USA (n=19), Canada (n=8), and South Africa (n=8; figure 2).

Study designs varied, with 76 studies attempting to include all culture-positive notified tuberculosis cases within the study period. 49 studies used a predefined subset of notified tuberculosis cases in the study region (eg, only individuals with multidrug-resistant tuberculosis were included). 12 studies included a random (or pseudorandom) sample of notified tuberculosis cases from the study region. Most studies did not restrict cases based on drug susceptibility, whereas 40 studies were specifically focused on investigating transmission among individuals with drug-resistant tuberculosis.

Although most studies included patients with tuberculosis detected through passive surveillance (eg, registered cases at health-care facilities), 34 studies incorporated cases identified through contact tracing. For example, studies in rural Uganda used registered individuals with tuberculosis and their contacts to identify locations of *M tuberculosis* transmission^14^ and areas of spatial overlap among cases.^15^ Walter and colleagues included individuals identified through active case findings from prisons in Brazil together with those identified in community settings to investigate the spillover of transmission from congregate settings to the surrounding population.^16^ The duration of collecting *M tuberculosis* isolates ranged from 6 months to 19 years. 47 studies included isolates collected for 2 years or less, 58 studies included isolates collected for 3–5 years, and 36 studies included isolates collected for periods exceeding 5 years.

### Spatially referenced genomic data

**Figure 3:**
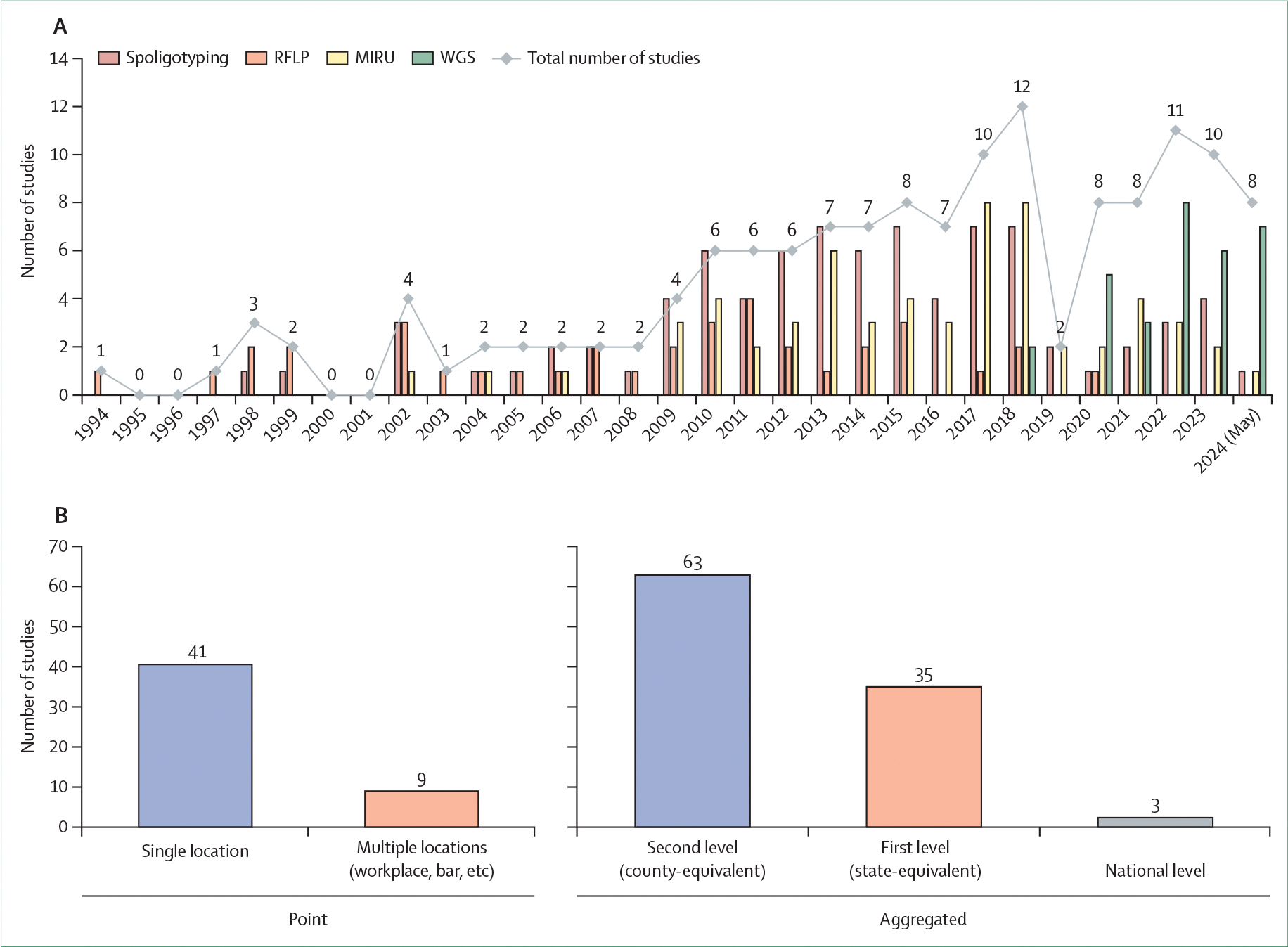
Summary of the included publications (A) Total number of studies (line) and number of studies by genotyping or genomic analysis methods used (bars) by year. (B) Spatial data by type and resolution. MIRU=mycobacterial interspersed repetitive unit. Spoligotyping=spacer oligonucleotide typing. WGS=whole-genome sequencing.

Genotyping and genomic sequencing methods varied across studies (figure 3A). Genotyping methods, including MIRU-VNTR analysis, spoligotyping, and RFLP, use categorical measures to define groups of genetically similar isolates that might be related through recent transmission. Conversely, WGS provides higher resolution by using discrete measures such as single nucleotide polymorphisms (SNPs) or continuous measures such as estimated transmission probabilities. In this Review, isolates assigned to a putative transmission cluster by either genotyping or sequencing approaches are referred to as members of an *M tuberculosis* cluster.

In 1994, Yang and colleagues used RFLP to identify three *M tuberculosis* clusters in Greenland and mapped their geographical locations.^17^ Since then, multiple genotyping and spatial analytical methods have been applied to investigate tuberculosis transmission, and the annual number of publications using genotyping methods was more than five-fold higher in 2018 than in 2010. However, the use of genotyping has declined with the increasing accessibility of genomic sequencing technologies. Two studies published in 2018 combined WGS with spatial analysis to investigate tuberculosis transmission,^18,19^ although WGS has been used in transmission studies since 2010.^20^ By 2020, sequencing had largely replaced lower-resolution genotyping techniques as the preferred genetic characterisation method (figure 3A).

### Spatial data types

More studies used areal data (ie, cases assigned to aggregated geographical regions; n=102) than point-referenced data (ie, cases assigned to unique spatial locations with coordinates; n=52). 12 studies used both point and aggregated data types and applied spatial methods to each. Among studies using areal data, the majority (n=64) assigned spatial locations of cases to small geographical areas (eg, county or equivalent level), whereas 35 studies aggregated cases at larger subnational scales (eg, state level). Three studies aggregated cases at the national level to examine *M tuberculosis* transmission across country borders.^21–23^

Among studies with point-referenced data, most (n=41) used a single location per participant (eg, primary residence), whereas nine studies included multiple locations per participant (eg, workplace, health-care facility, or other places of social congregation). For example, one study examined potential *M tuberculosis* transmission sites located near a major railway in an urban setting in Japan,^24^ and another study in Benin identified individuals who lived or worked in the same area and visited the same bar and were infected with a closely related *M tuberculosis* Beijing isolate.^25^ A study in Lima, Peru, constructed spatial activity spaces using GPS tracking and identified overlapping activity spaces among individuals infected with genetically similar multidrug-resistant *M tuberculosis* isolates.^26^

### Research questions addressed using genomic and spatial data

Studies included in this Review addressed four general types of research questions. The first category, spatial description (a), addressed how individuals with tuberculosis and specific *M tuberculosis* clusters are geographically distributed across study areas. The second category, hotspot detection and localisation (b), investigated whether locations of spatial concentration of specific *M tuberculosis* clusters exist and, in such cases, where these areas are located. The third category, hotspot explanation (c), assessed whether host, pathogen, or environmental factors contribute to these spatial patterns. The final category, association between proximity and genetic relatedness (d), explored whether relationships existed between the spatial proximity of individuals with tuberculosis and the genetic similarity of the *M tuberculosis* isolates with which they were infected.

The resolution of spatial and genetic data determined which research questions could be addressed, and many studies attempted to examine more than one of these questions. From a practical standpoint, all studies meeting the inclusion criteria addressed at least the first question. A table of all included studies, categorised by research question and methods used, is provided in the appendix (pp 10–15; the relevant reference list is in the appendix [pp 16–25]).

Among the studies addressing spatial description (question a), 89 provided only simple descriptions of the spatial distribution of *M tuberculosis* clusters. Of these, 21 studies provided spatial information solely through text or tables, such as listing genetically typed cases by administrative region (eg, state or province) of primary residence. Studies that included maps (n=57) primarily used dot maps to display point data (typically after jittering to maintain privacy), and pie charts and choropleth maps were used to visualise areal data.

Beyond simple description, 32 studies included statistical tests to assess associations between administrative areas and the locations of genetically clustered isolates. For example, a study in New Jersey, USA, found that individuals with tuberculosis who reside in a particular county were at higher risk of being in a specific *M tuberculosis* genotype cluster.^27^ Other studies have grouped all genotypically linked cases as a single category and compared their spatial distribution to that of all other cases; for example, a study in South Africa indicated that individuals living in urban districts have a higher probability of being infected with genetically similar *M tuberculosis* strains than those living in rural districts.^28^

In studies focused on hotspot detection and localisation (question b), 46 attempted to identify specific areas of spatial aggregation of *M tuberculosis* clusters. Most studies seeking to identify areas of unexpectedly high spatial aggregation accounted for variations in local population density. Some explicitly incorporated population per area in their analyses, whereas others implicitly controlled for population density by comparing the spatial distributions of individuals infected with different *M tuberculosis* strains.

Studies used various methods to detect and localise *M tuberculosis* clusters depending on the type of spatial data available. Spatial scan analysis was the most frequently used approach (n=21) for investigating the spatial aggregation of cases. A spatial scan statistic tests for evidence of clustering within circular or elliptical windows.^29^ The scan statistic has been used for detecting spatial concentration of *M tuberculosis* clusters since 2007^30^ and has been widely applied to tuberculosis^8^ and other diseases.^31^

Spatial autocorrelation methods were applied in studies using aggregated data and facilitated the investigation of clustering patterns at both global and local levels. Two studies used Global Moran’s *I* to test for evidence of any significant spatial aggregation of *M tuberculosis* clusters across entire study areas.^32^ Local methods such as the Getis-Ord Gi* statistic were used to identify hotspots and coldspots based on local estimates of spatial autocorrelation.^33,34^ Four studies in our scoping review used these methods to identify hotspots of *M tuberculosis* clusters, providing evidence of localised transmission.

13 studies used density-based methods to identify areas of spatial aggregation, with kernel density estimation the most common method, being used in 11 of these studies. These methods help to estimate the intensity of cases within grid cells covering the study area.

Distance-based methods were used in four studies, applying nearest neighbour analysis^31^ to evaluate whether spatial clustering occurred in relation to each case and its *k*^th^ nearest neighbour. Three studies used Ripley’s *K* function^35^ to estimate the relationship between genetic clustering and spatial distance, whereas two studies used distance-based mapping through a case-control approach, in which cases (isolates within a specific transmission network) were compared with controls (isolates outside the transmission network).^36^ One study used a spatial Bayesian model to identify local foci of tuberculosis transmission.^37^ Although this study used areal data, Bayesian models can be adapted for use with point data.

Hotspot explanation (question c) was addressed by 11 studies that examined factors associated with the spatial concentration of *M tuberculosis* clusters using hypothesis testing and regression modelling. We illustrate this approach with a simple regression model. The outcome specific to the individual or spatial unit i (ie, $Y_{i}$) can take several forms; it might be a binary indicator (eg, being a member of a genomic cluster or not), a count (eg, the number of cases belonging to a particular genomic cluster within a spatial unit), or other possible outcomes depending on the research question. The vector of corresponding covariates (ie, $x_{i}$) can include a mix of host characteristics (eg, age), environmental factors (eg, population density), or *M tuberculosis* isolate-related information (eg, lineage). An example of a spatial regression model for a continuous outcome is expressed as.

$$Y_{i}=\beta_{0}+\sum_{j=1}^{p}\beta_{j}x_{ij}+\theta_{i}+\epsilon_{i}$$

in which the $\beta_{j}(j>0)$ parameters describe the association between the predictors and outcome, and $\theta_{i}$ is a random effect that can be included (but is not required) to account for spatial correlation in the outcome data. The distributional assumptions of spatial random effects depend on the type of spatial data and other sources of aggregation (eg, individuals within a household). Geostatistical methods based on Gaussian processes are appropriate for point-referenced data, whereas conditional autoregressive models better describe spatial proximity and correlation in areal data.^37–39^ The term $\epsilon_{i}$ is a typical error term that accounts for data distribution but is often omitted in non-continuous outcomes (eg, binary or count data).

For example, a study in Botswana used scan statistics to identify areas of localised *M tuberculosis* transmission. A subsequent multivariable logistic regression analysis helped to identify host-specific factors (ie, age <24 years, smoking, and unemployment) and environmental factors (ie, residence in an area with high tuberculosis incidence) that were associated with increased risk of being in a local transmission hotspot.^40^

A study in Moldova indicated that local population density was positively associated with hotspot locations of *M tuberculosis* clusters, whereas factors such as local tuberculosis incidence and local measures of poverty were not associated with transmission hotspots.^37^ Notably, spatial regression modelling also allows the integration of additional covariates into spatial aggregation analyses, enabling researchers to address both cluster detection and explanatory factors within a single analytical framework.

The association between proximity and genetic relatedness (question d) was examined in 15 studies that explored the correlation between genetic distance between *M tuberculosis* isolates (ie, a measure of pathogen relatedness) and the spatial proximity of tuberculosis cases. One study in Shanghai, China, reported that individuals with tuberculosis living within 1 km of each other had the highest risk of being infected with closely related *M tuberculosis* strains, indicating that household proximity was associated with transmission.^41^ Pairwise analyses have been used to investigate the correlation between geographical distance and genetic relatedness. For example, a study in Botswana indicated generally low positive correlations between pairwise proximity and SNP distances, although the strength of the correlation varied by genotype cluster.^42^ Methods that account for multiple sources of correlation while testing for the association between covariates and genetic similarity between *M tuberculosis* isolate pairs, such as GenePair,^43^ have also been introduced. The methods that have been widely used for addressing these four types of research questions with point and areal data are listed in table 1.

### Impact of the pathogen-typing approach on research questions and analytical methods

Different research questions have been addressed, and distinct analytical methods have been used, depending on whether genotyping (n=111) or sequencing (n=31) approaches were used.

In studies using pathogen genotyping methods such as RFLP, MIRU-VNTR, or spoligotyping, approximately 70% (n=77 of 111) focused on describing the spatial distribution of specific *M tuberculosis* isolates (question a). 33 of these studies extended the analysis to investigate the locations of spatial aggregation of *M tuberculosis* clusters (question type b) and nine further investigated factors associated with such hotspots (question type c). Given that genotyping typically classifies *M tuberculosis* isolates into nominal categories, only two genotyping studies attempted to estimate the relationship between genetic distance and spatial proximity (question d).

In contrast, studies using WGS adopted more advanced approaches to integrate genomic and spatial data. 12 WGS studies focused on describing the spatial distributions of *M tuberculosis* isolates (question a), whereas 19 studies used more sophisticated methods to analyse genomic and spatial data (table 2). Of these, 13 identified areas of spatial aggregation of genomically classified *M tuberculosis* isolates (question b) and two performed additional analyses to identify factors associated with these hotspots (question c). Furthermore, 13 WGS studies quantified the association between genomic and spatial distances of isolate pairs (question d).

WGS studies offer unique opportunities for defining genomic clustering and identifying potential transmission linkages. Two studies relied solely on common (sub)lineage assignments to define genomic clusters, whereas 29 studies used thresholds of SNP distance (or related estimates) to define transmission links. The most commonly applied threshold was 12 SNPs (n=13), followed by thresholds of five SNPs (n=10), ten SNPs (n=6), 11 SNPs (n=1), and 20 SNPs (n=1). Two studies used multiple SNP thresholds in their main analysis, whereas 27 studies used a single threshold for the main analysis and conducted sensitivity analyses with alternate thresholds. Additionally, some WGS studies used estimated patristic distance (n=3) or estimated transmission probability (n=6) as alternative continuous measures of genomic similarity. The studies integrating WGS with spatial analysis are summarised in table 2.

## Discussion

In our scoping review, 142 studies on *M tuberculosis* combining genomic and spatial data met the inclusion criteria. Most studies were conducted in North America and Asia, with fewer studies from South America and Africa. This disparity likely reflects uneven access to genomic sequencing resources and suggests that knowledge of transmission patterns in some of the most affected countries remains scarce. Most studies included tuberculosis cases detected through routine passive surveillance over study durations of 5 years or less. Approximately one-quarter of studies focused specifically on drug-resistant tuberculosis and several relied on data collected during surveys conducted for other purposes. Over the past 5 years, genotyping methods such as RFLP, MIRU-VNTR, and spoligotyping have largely been replaced by WGS for characterising pathogen relatedness and inferring transmission, with important implications for the types of research questions that can be addressed and the analytical methods used.

Studies included in this Review contained descriptions of the spatial locations associated with specific *M tuberculosis* clusters, often presenting maps using dot plots or choropleth maps, depending on the available resolution of the spatial data and non-graphical tabular descriptions (question a). Many studies incorporating WGS methods have also displayed additional data beyond the categorical *M tuberculosis* cluster types on these maps, such as integrating spatial locations with phylogenetic trees.^42,56^

Beyond the descriptive mapping of specific *M tuberculosis* isolates, most studies included additional analyses to test for evidence of spatial aggregation (question b). The selection of the most appropriate methods to assess spatial clustering depends on the resolution of the spatial data (areal *vs* point). Available methods to test for these types of hotspots include approaches for the detection of spatial autocorrelation (eg, Getis-Ord Gi*), methods for the detection of spatial aggregation (eg, scan analysis), density-based approaches (eg, kernel density estimation), and distance-based methods (eg, distance-based mapping). The application of spatial modelling approaches (eg, hierarchical Bayesian modelling) has been increasingly used to detect spatial aggregation, and these methods allow for the inclusion of additional covariates that might be associated with spatial aggregation (question c). Properly accounting for local population density differences when identifying areas with a higher-than-expected number of tuberculosis cases within specific *M tuberculosis* clusters remains an important consideration for researchers.

Many studies that identified tuberculosis hotspots also sought to identify host, pathogen, and environmental factors associated with spatial aggregation (question c). The existing literature includes studies that use both spatial and non-spatial models; we strongly encourage researchers to consider using models that account for spatial autocorrelation to ensure valid statistical inference when working with spatially structured data.

WGS has provided additional opportunities to investigate the association between genomic relatedness and spatial distance among *M tuberculosis* isolates (question d). These studies have typically used SNP differences as a measure of genetic relatedness (table 2), but tree-based measures (eg, patristic distances) or estimates obtained from formal transmission inference (eg, transmission probabilities) are increasingly used;^57^ these approaches can account for other measured variables or features of the data such as censoring and incomplete sampling. Pairwise regression analyses, in which genetic distance and spatial distance between each pair of sequenced *M tuberculosis* isolates are evaluated, are commonly used to investigate these relationships. However, these approaches require specific analytical methods to account for correlations introduced by pairwise comparisons, as each isolate appears in multiple pairs.^58^ GenePair^43^ provides a Bayesian approach for analysing these data while incorporating other measured covariates.

Several limitations should be considered when interpreting the findings of this Review. First, only articles published in English were included, which might have led to the omission of relevant studies published in other languages. Second, we did not evaluate the quality of the included studies. Third, we did not provide a detailed technical review of the genetic and spatial epidemiological methods used; we refer interested readers to relevant reviews of these topics.^8,59^

As genomic and spatial analytical methods continue to evolve, we anticipate numerous opportunities and challenges in the future. The measurement of spatial locations can be challenging in many settings, and new technologies and approaches for assigning accurate locations will be valuable. The topic of measurement errors in spatial analyses has received attention elsewhere.^60^ This issue is particularly relevant for many locations with high tuberculosis burden where the automated conversion of street addresses to spatial coordinates (ie, geocoding) remains unfeasible. Most studies use residential addresses as the primary spatial location, whereas some also collect data on additional locations, including workplaces, schools, places of worship, transportation hubs, prisons, and health-care facilities. Identifying the most appropriate approach for incorporating multiple locations remains an area of active investigation. Given the increasing recognition of the importance of transmission occurring outside households,^61^ and in the context of other congregate settings,^62^ new methods for identifying shared locations and transmission-prone environments will be valuable. Furthermore, most included studies used Euclidean or other geographical distances, which might not adequately capture transmission-relevant connectivity between locations.^63^ The use of mobility data, which are becoming increasingly common in infectious disease epidemiology, as shown in studies on COVID-19^64,65^ and malaria,^66^ presents promising avenues for addressing this limitation.^67,68^

A key challenge that limits transmission inference relates to incomplete sampling of transmission networks. This limitation occurs for several reasons, only some of which are modifiable by investigators. First, epidemics of *M tuberculosis* progress more slowly than those of most other pathogens, resulting in the left-censoring and right-censoring in epidemiological studies. Conducting studies over longer timeframes might help to partly address this issue, but we anticipate that such censoring would be persistent. Second, a substantial proportion (approximately 40% globally) of notified tuberculosis cases are diagnosed without microbiological confirmation; thus, isolates of *M tuberculosis* infections are often unavailable for sequencing. The optimal approach for handling untypable cases in transmission analyses remains unclear and likely depends on the specific study setting and research question. Some efforts have been made to predict the *M tuberculosis* cluster to which untyped isolates would have been assigned in a low-incidence setting,^69^ but whether these predictions are sufficiently accurate and whether these methods generalise to high-incidence settings are unclear. Third, the prevalence of asymptomatic tuberculosis is increasingly recognised; surveys assessing tuberculosis prevalence have shown that approximately 50% of patients with prevalent culture-positive tuberculosis do not report symptoms typically associated with this disease.^70^ Our understanding of the natural history of asymptomatic disease (ie, what subset of asymptomatic individuals would have developed symptoms, sought care, and at what time) and its contribution to transmission is incomplete; nevertheless, the presence of such asymptomatic cases complicates the interpretation of the data that are typically available.

Another common challenge is that many *M tuberculosis* clusters contain a small number of cases, making statistical inference difficult. Restricting analyses to larger genetic clusters (eg, those with at least ten cases) can mitigate some of these issues; however, this approach risks overlooking important insights into the early growth and spread of clusters and might exclude a substantial proportion of the available data. Assuming shared characteristics across clusters, hierarchical modelling that incorporates all clusters within a unified framework could facilitate the pooling of information and improve the stability of inference across clusters of varying sizes. Meta-analyses and meta-regressions might also be valuable for integrating information from cluster-specific analyses while accounting for size differences by incorporating uncertainty measures into the analysis.

We anticipate that novel methods for integrating, visualising, and analysing the rich spatial and geographical information that are now available will be introduced in future studies. Machine learning and artificial intelligence-based approaches for combining spatial and genomic data are under development; however, no peer-reviewed studies using these methods were identified in our search. The development of accessible web-based tools that combine spatial and genomic data to enable more rapid assessments of local transmission locations would serve as a valuable resource for policy makers, supporting more effective and efficient resource allocation. In all cases, preserving patient anonymity and protecting affected communities will be essential to ensure that the potential benefits of these tools are realised without compromising care or increasing stigma.

## Supplementary Material

1

## Figures and Tables

**Table 1:** Methods used to address the four types of questions with point or areal data

	Question a (spatial description)	Question b (hotspot detection and localisation)	Question c (hotspot explanation)	Question d (association between proximity and genetic relatedness)

**Point data**	Dot map (eg, using colour or shape to represent different *Mycobacterium tuberculosis* clusters) Illustration using text in a table Hypothesis testing (eg, ANOVA)	Density-based methods (eg, kernel density estimation) Neighbour-based methods (eg, nearest neighbour index) Distance-based methods between cases and controls (eg, distance-based mapping) Spatial (Bayesian) modelling	Hypothesis test (eg, ANOVA) Regression modelling Geostatistical spatial modelling	Correlation Regression modelling Spatial (Bayesian) modelling
**Areal data**	Display aggregated information at the centroid of the locality (eg, pie chart and graduated symbol) Choropleth map Illustration using text in a table Hypothesis test (eg, χ^2^ test)	Global and local spatial autocorrelation (eg, Global Moran’s *I* and Getis-Ord Gi*) Scan statistics Spatial (Bayesian) modelling	Hypothesis test (eg, χ^2^ test) Regression modelling Disease mapping	Correlation Regression modelling Spatial (Bayesian) modelling

**Table 2:** Studies combining whole-genome sequencing and spatial data to address research questions beyond spatial distribution

	Study duration	Study area	Definition of genomic clusters	Spatial approaches	Types of questions addressed	Focus on DR-TB

Yang et al (2018)^19^	2009–15	Shanghai, China	SNP (10) and transmission probability	KDE	a, b, d	··
Jiang et al (2020)^44^	2013–17	Shenzhen, China	SNP (12)	KDE	a, b	✓
Lin et al (2020)^45^	2018	Guangxi, China	SNP (12)	NA	a, d	··
Bui et al (2021)^26^	2016–17	Lima, Peru	SNP (5)	KDE	a, b, c, d	✓
Huang et al (2022)^46^	2009–12	Lima, Peru	SNP (1, 5, 10)	Other	a, b, d	··
Yin et al (2022)^47^	2018–20	Beijing, China	SNP (12) and transmission probability	KDE	a, b	✓
Yang et al (2022)^48^	2018–19	Moldova	SNP (5), patristic distance, and transmission probability	NA	a, d	··
Zhao et al (2022)^49^	2018–20	Chongqing, China	SNP (12)	NA	a, d	✓
Baker et al (2023)^42^	2012–16	Gaborone, Botswana	SNP (5)	KDE, K function	a, b, d	··
Li et al (2023)^41^	2011–20	Shanghai, China	SNP (12) and transmission probability	KDE	a, b, d	··
Miyahara et al (2023)^50^	2017–20	Chiang Rai province, Thailand	SNP (12)	Nearest neighbour index	a, b	··
Che et al (2024)^51^	2020–23	Ningbo, China	SNP (12)	KDE	a, b, d	✓
Lan et al (2024)^37^	2018–19	Moldova	SNP (5), patristic distance, and transmission probability	Spatial Bayesian modelling	a, b, c	··
Liu et al (2024)^52^	2015–21	Zhejiang, China	SNP (12)	KDE	a, b, d	··
Utpatel et al (2024)^53^	2017–19	Callao, Peru	SNP (5)	KDE	a, b	✓
Yang et al (2024)^54^	2016–21	Urumqi City, China	SNP (12)	Scan statistics	a, b	✓
Yuen et al (2024)^55^	2011–12 and 2020–21	Peru	SNP (5, 10)	NA	a, d	··

A checkmark in the DR-TB column indicates a study that focuses on at least one drug-resistant phenotype, including multidrug-resistant and extensively drug-resistant phenotypes. DR-TB=drug-resistant tuberculosis. KDE=kernel density estimation. NA=not applicable. SNP=single nucleotide polymorphism. Question a=spatial description. Question b=hotspot detection and localisation. Question c=hotspot explanation.

Question d=association between proximity and genetic relatedness.
