# Supplementary material for: Integrating genomic and spatial analyses to describe tuberculosis transmission: a scoping review
Source: Lancet Microbe. Author manuscript; Available in PMC 2025 Aug 22. (PMC12371702; doi:10.1016/j.lanmic.2025.101094)
Supplement: 1 [file NIHMS2102286-supplement-1.pdf]

THE LANCET
Microbe

Supplementary appendix

This appendix formed part of the original submission and has been peer reviewed.
We post it as supplied by the authors.

Supplement to: Lan Y, Rancu I, Chitwood MH, et al. Integrating genomic and spatial analyses to describe tuberculosis transmission: a scoping review. *Lancet Microbe* 2025. https://doi.org/10.1016/j.lanmic.2025.101094

Query strings of four searched databases

PubMed: (("Genotyping Techniques"[MeSH Terms] OR "geno*"[Title/Abstract] OR "whole genome sequencing"[MeSH Terms:noexp] OR "whole genome sequencing"[Title/Abstract] OR "WGS"[Title/Abstract]) AND ("tuberculosis"[MeSH Terms] OR "tuberculosis"[Title/Abstract] OR "TB"[Title/Abstract]) AND ("spatial analysis"[MeSH Terms] OR "geographic information systems"[MeSH Terms] OR "spatial*"[Title/Abstract] OR "spatio*"[Title/Abstract] OR "geographic*"[Title/Abstract] OR "heterogeneous distribution*"[Title/Abstract] OR "spacetime cluster*"[Title/Abstract] OR "space time cluster*"[Title/Abstract] OR "hotspot*"[Title/Abstract] OR "hot spot*"[Title/Abstract] OR "geospatial*"[Title/Abstract])) NOT ("animals"[MeSH Terms] NOT "humans"[MeSH Terms])

Web of Science Core Collection: TS=((("Genotyping Techniques" OR geno* OR "whole genome sequencing" OR "whole genome sequencing" OR WGS) AND (tuberculosis OR tuberculosis OR TB) AND ("spatial analysis" OR "geographic information systems" OR spatial* OR spatio* OR geographic* OR "heterogeneous distribution*" OR "spacetime cluster*" OR "space time cluster*" OR hotspot* OR "hot spot*" OR geospatial*)) NOT (animals NOT humans))

EMBASE Ovid: ((exp "Genotyping Techniques"/ OR geno*.tw. OR "whole genome sequencing"/ OR "whole genome sequencing".tw. OR WGS.tw.) AND (exp tuberculosis/ OR tuberculosis.tw. OR TB.tw.) AND (exp "spatial analysis"/ OR exp "geographic information systems"/ OR spatial*.tw. OR spatio*.tw. OR geographic*.tw. OR "heterogeneous distribution*".tw. OR "spacetime cluster*".tw. OR "space time cluster*".tw. OR hotspot*.tw. OR "hot spot*".tw. OR geospatial*.tw.)) NOT (exp animals/ NOT exp humans/)

Scopus: TITLE-ABS-KEY (("Genotyping Techniques") OR (geno*) OR ("whole genome sequencing") OR (wgs) AND ((tuberculosis) OR (tb)) AND (("spatial analysis") OR ("geographic information systems") OR (spatial*) OR (spatio*) OR (geographic*) OR ("heterogeneous distribution*") OR ("spacetime cluster*") OR ("space time cluster*") OR (hotspot*) OR ("hot spot*") OR (geospatial*))) AND (LIMIT-TO (LANGUAGE , "English"))

Extraction template

Extract information	Details or example
**Title**	
**Published Year**	
**Study design and setting**	e.g., whether the study was a convenience sample or really tried to get most cases from a geographic area. And whether all cases were sequenced or just a subset meeting some criteria (ie MDR, etc)
**Country that the study conducted**	include all the county name if the study includes more than one country.
**Data source**	e.g., register TB cases, survey, active finding cases, etc.
**TB incidence of the study area**	it could be the number, or description (e.g., high incidence area).
**Sampling procedures**	details of the proportion of sequenced data: whole or a subset; reason
**Number of total TB cases**	
**Location representation**	e.g., individual residence, clinic, village, grid
**Unit of analysis**	e.g., location (residence or clinic), village, census tract, grid, etc.
**Study scale**	e.g., city, state, country, world
**Study time range (start time and end time)**	e.g., 2014-2020
**Genomic analysis methods**	e.g., spoligotyping, MIRU, RFLP and WGS
**Spatial analysis methods**	e.g., SaTScan, mapping, etc.
**The way of integrating two methods**	e.g., yes, they applied spatial methods to genotype clusters; Or no integration
**Definition of genetic "cluster"**	The definition used to define a “cluster” of genetically related strains (i.e., the SNP cutoff used or something similar for WGS study; or exact matches or one band difference for MIRU or spoligotype or RFLP).
**The use of the term "cluster" for spatial aggregation**	Yes or no, this extract is aimed to record any confusion on using the "cluster" term for both genetic and spatial aspect.
**Main finding in terms of transmission pattern**	e.g., they found spatial aggregation of cases from genomic clusters

Pathogen genetic characterization

Three genotyping methods plus are used to identify genetic differences in *Mycobacterium tuberculosis* (*M.tb*):

Spoligotyping (Spacer oligonucleotide typing): This PCR-based technique analyzes a specific region of the *M.tb* genome called the direct repeat region (DR). The DR region contains multiple copies of a 36 bp sequence, separated by non-repetitive spacers – the presence (1) or absence (0) of spacers.

RFLP (Restriction Fragment Length Polymorphism): This method analyzes DNA fragments created by a restriction enzyme which cuts at specific DNA sequences, followed by use of an Insertion Sequence probe (6110) which allows for identification of variations in number and size of fragment lengths.

MIRU (Mycobacterial Interspersed Repetitive Unit Variable Number Tandem Repeats): This PCR-based method utilizes PCR to analyze repetitive DNA sequences called MIRU; the most commonly used version of this approach tests for variable numbers of tandem repeats at 24 loci.

WGS (Whole Genome Sequencing): This method sequences nearly the entire genome of *M.tb*, providing the most detailed information about genetic variation throughout the genome; highly repetitive regions, such as the PE/PPE region are often not included in analyzed regions of the genome.

Table S1. Included studies that integrated genomic and spatial analyses to understand tuberculosis transmission.

Article ID	Title	Year	Type of Funding	Funding Source
1	Restriction fragment length polymorphism of Mycobacterium tuberculosis strains isolated from Greenland during 1992: Evidence of tuberculosis transmission between Greenland and Denmark[1]	1994	Government	International
2	Restriction fragment length polymorphism of Mycobacterium tuberculosis strains from various regions of India, using direct repeat probe[2]	1997	Government	National
3	Diversity of DNA fingerprints of Mycobacterium tuberculosis isolates in the United States[3]	1998	Government	National
4	Spoligotypes of Mycobacterium tuberculosis complex isolates from patients residents of 11 states of Brazil[4]	2012	University	National
5	Conspicuous multidrug-resistant Mycobacterium tuberculosis cluster strains do not trespass country borders in Latin America and Spain[5]	2012	Industry and Government	National
6	Comparative Mycobacterium tuberculosis spoligotype distribution in Mexico[6]	2014	Government	National

7	Genetic diversity and drug susceptibility profile of Mycobacterium tuberculosis isolated from different regions of India[7]	2015	Government	National
8	Molecular epidemiology of Mycobacterium tuberculosis in Baja California, Mexico: A result of human migration?[8]	2017	Government	National
9	Analysis of mycobacterium tuberculosis genotypic lineage distribution in Chile and neighboring countries[9]	2016	na	na
10	Pulmonary tuberculosis in Harare, Zimbabwe: analysis by spoligotyping[10]	1998	University	National
11	Spatial Patterns of Extensively Drug-Resistant Tuberculosis Transmission in KwaZulu-Natal, South Africa[11]	2018	Government	International
12	Genomic epidemiology of Mycobacterium tuberculosis in Santa Catarina, Southern Brazil[12]	2020	Government	National
13	Dissemination of Mycobacterium tuberculosis across the San Francisco Bay Area[13]	1998	Government	National
14	Molecular epidemiology of tuberculosis in Malaysia[14]	1999	Government, Industry	International
15	Evidence from molecular fingerprinting of limited spread of drug- resistant tuberculosis in Texas[15]	1999	Government	National
16	IS6110 fingerprinting of sensitive and resistant strains (1991-1992) of Mycobacterium tuberculosis in Colombia[16]	2002	na	na
17	Transmission of drug-resistant tuberculosis in Texas and Mexico[17]	2002	Non-profit organizations	National
18	Identification and evolution of an IS6110 low-copy-number Mycobacterium tuberculosis cluster[18]	2002	Government	National
19	Historic and recent events contribute to the disease dynamics of Beijing-like Mycobacterium tuberculosis isolates in a high incidence region[19]	2002	Industry and Government	National
20	Clonal expansion of multidrug-resistant and extensively drug-resistant tuberculosis, Japan[20]	2010	Government	National
21	Mycobacterium tuberculosis cluster with developing drug resistance, New York, New York, USA, 2003-2009[21]	2011	Government	National
22	Identifying location of recent TB transmission in rural Uganda: A multidisciplinary approach[22]	2014	Government	International
23	Spatial overlap links seemingly unconnected genotype-matched TB cases in rural Uganda[23]	2018	Government	International
24	A complex scenario of tuberculosis transmission is revealed through genetic and epidemiological surveys in Porto[24]	2018	Government	Mixed
25	Molecular epidemiology and genetic diversity of Mycobacterium tuberculosis complex in referral health centers of Bamako, Mali: What is new?[25]	2022	Government	National
26	Spatial Distribution of Drug-Resistant Mycobacterium tuberculosis Infections in Rural Eastern Cape Province of South Africa[26]	2023	Government	National
27	Conventional and molecular epidemiology of Tuberculosis in Manitoba[27]	2003	na	na
28	A dominant Mycobacterium tuberculosis strain emerging in Denmark[28]	2004	na	na
29	Geo-Epidemiologic and Molecular Characterization to Identify Social, Cultural, and Economic Factors Where Targeted Tuberculosis Control Activities Can Reduce Incidence in Maryland, 2004-2010[29]	2013	na	na
30	Clustering of Mycobacterium tuberculosis Cases in Acapulco: Spoligotyping and Risk Factors[30]	2011	Government	National
31	Neighborhood socioeconomic position and tuberculosis transmission: a retrospective cohort study[31]	2014	na	na

32	Molecular Epidemiology and Genetic Diversity of Multidrug-Resistant Mycobacterium tuberculosis Isolates in Bangladesh[32]	2022	Government	International
33	Detecting tuberculosis clusters in urban neighborhoods, Taipei, Taiwan: Linking geographic and genotyping evidence[33]	2019	Government, University	National
34	Internal migration and transmission dynamics of tuberculosis in Shanghai, China: an epidemiological, spatial, genomic analysis[34]	2018	Government	Mixed
35	Using statistical methods and genotyping to detect tuberculosis outbreaks[35]	2013	Government	National
36	Using routinely reported tuberculosis genotyping and surveillance data to predict tuberculosis outbreaks[36]	2012	Government	National
37	Using genotyping and geospatial scanning to estimate recent mycobacterium tuberculosis transmission, United States[37]	2012	na	na
38	High rates of clustering of strains causing tuberculosis in Harare, Zimbabwe: a molecular epidemiological study[38]	2004	University, non-profit	International
39	Tuberculosis and homelessness in Montreal: a retrospective cohort study[39]	2011	Government	National
40	Transmission pattern of drug-resistant tuberculosis and its implication for tuberculosis control in eastern rural China[40]	2011	Government	National
41	Transmission of multidrug-resistant tuberculosis in Shanghai: roles of residential status[41]	2018	Government, University	Mixed
42	Three-year longitudinal study of genotypes of Mycobacterium tuberculosis in a low prevalence population[42]	2010	Government	National
43	Spoligotypes of Mycobacterium tuberculosis from different Provinces of China[43]	2010	Government	National
44	Spoligotype-based comparative population structure analysis of multidrug-resistant and isoniazid-monoresistant Mycobacterium tuberculosis complex clinical isolates in Poland[44]	2010	Government	National
45	Spatiotemporal Clustering of Mycobacterium tuberculosis Complex Genotypes in Florida: Genetic Diversity Segregated by Country of Birth[45]	2016	University	National
46	Spatio-Temporal Distribution of Mycobacterium tuberculosis Complex Strains in Ghana[46]	2016	Non-profit organizations	International
47	Spatial-temporal distribution of genotyped tuberculosis cases in a county with active transmission[47]	2017	na	na
48	Phylogeography and transmission of M. tuberculosis in Moldova: A prospective genomic analysis[48]	2022	Government	International
49	Spatial clustering and genetic diversity of Mycobacterium tuberculosis isolate among pulmonary tuberculosis suspected patients, Arsi Zone, Ethiopia[49]	2021	University	National
50	Citywide Transmission of Multidrug-resistant Tuberculosis Under China's Rapid Urbanization: A Retrospective Population-based Genomic Spatial Epidemiological Study[50]	2020	Government, non-profit, university	Mixed
51	Socio-demographic and geographic indicators and distribution of tuberculosis in Hong Kong: A spatial analysis[51]	2005	Government	National
52	Geno-Spatial Distribution of Mycobacterium Tuberculosis and Drug Resistance Profiles in Myanmar-Thai Border Area[52]	2020	Government, University	Mixed
53	Distribution of the Beijing family genotypes of Mycobacterium tuberculosis in Taiwan[53]	2005	Government	National
54	Prioritizing tuberculosis clusters by genotype for public health action, Washington, USA[54]	2013	na	na
55	Epidemiology characteristics of the clonal complexes of Mycobacterium tuberculosis Lineage 4 in China[55]	2020	Government	National

56	Population-Based Geospatial and Molecular Epidemiologic Study of Tuberculosis Transmission Dynamics, Botswana, 2012-2016[56]	2021	Government	International
57	Community transmission of multidrug-resistant tuberculosis is associated with activity space overlap in Lima, Peru[57]	2021	Non-profit organizations	International
58	Genomic and geospatial epidemiology of Mycobacterium tuberculosis in Oman: first national insight using whole genome sequencing[58]	2023	Government	National
59	Phylogeographical particularity of the Mycobacterium tuberculosis Beijing family in South Korea based on international comparison with surrounding countries[59]	2010	Government	Mixed
60	Phylogenetic diversity of Mycobacterium tuberculosis in two geographically distinct locations in Botswana - The Kopanyo Study[60]	2020	na	na
61	Outbreak of tuberculosis among substance users and homeless people in Greater Montréal, Canada, 2003-2016[61]	2017	na	na
62	The Mycobacterium tuberculosis Uganda II family and resistance to first-line anti-tuberculosis drugs in Uganda[62]	2014	Government	International
63	Long-term population-based genotyping study of Mycobacterium tuberculosis complex isolates in the French departments of the Americas[63]	2006	Government	Mixed
64	Mycobacterium tuberculosis complex genotypes circulating in Nigeria based on spoligotyping obtained from Ziehl-Neelsen stained slides extracted DNA[64]	2018	Government	International
65	Mycobacterium tuberculosis Beijing genotype is associated with HIV infection in Mozambique[65]	2013	University	Mixed
66	Mycobacterium africanum is associated with patient ethnicity in Ghana[66]	2015	Government, non-profit	Mixed
67	Molecular, Spatial, and Field Epidemiology Suggesting TB Transmission in Community, Not Hospital, Gaborone, Botswana[67]	2017	na	na
68	Molecular epidemiology of tuberculosis in the Czech Republic, 2004: analysis of M. tuberculosis complex isolates originating from the city of prague, south Moravia and the Moravian-Silesian region[68]	2006	na	na
69	Molecular epidemiology of tuberculosis in Cambodian children[69]	2015	University, non-profit	National
70	Molecular Epidemiology of Mycobacterium tuberculosis strains isolated from pulmonary tuberculosis patients in south Ethiopia[70]	2021	Government	National
71	Molecular epidemiology of Mycobacterium tuberculosis in aboriginal peoples of Taiwan, 2006-2011[71]	2014	Government	National
72	Use of geographic and genotyping tools to characterise tuberculosis transmission in Montreal[72]	2007	Government, University	National
73	Local epidemic history as a predictor of tuberculosis incidence in Saskatchewan Aboriginal communities[73]	2011	Government	International
74	Investigation on Mycobacterium tuberculosis diversity in China and the origin of the Beijing clade[74]	2011	Government	National
75	Investigating extradomiciliary transmission of tuberculosis: An exploratory approach using social network patterns of TB cases and controls and the genotyping of Mycobacterium tuberculosis[75]	2020	na	na
76	Identifying Hotspots of Multidrug-Resistant Tuberculosis Transmission Using Spatial and Molecular Genetic Data[76]	2016	Government, non-profit	International
77	Identifying areas and risk groups with localised Mycobacterium tuberculosis transmission in northern England from 2010 to 2012: spatiotemporal analysis incorporating highly discriminatory genotyping data[77]	2016	na	na
78	Foreign-Born Status and Geographic Patterns of Tuberculosis Genotypes in Tarrant County, Texas[78]	2007	Government	National
79	Clusters of drug-resistant mycobacterium tuberculosis detected by whole-genome sequence analysis of nationwide sample, Thailand, 2014–2017[79]	2021	Government	National
80	HIV infection and geographically bound transmission of drug-resistant tuberculosis, Argentina[80]	2012	Government	National

81	Geography and genealogy of the human host harbouring a distinctive drug-resistant strain of tuberculosis[81]	2008	Government, University	National
82	Genotypic diversity of extensively drug-resistant tuberculosis (XDR-TB) in South Africa[82]	2008	Government, University	National
83	High diversity of multidrug-resistant Mycobacterium tuberculosis Central Asian Strain isolates in Nepal[83]	2017	Government	International
84	Genotyping and spatial analysis of pulmonary tuberculosis and diabetes cases in the state of Veracruz, Mexico[84]	2018	Government	National
85	Genotyping and drug resistance patterns of Mycobacterium tuberculosis strains observed in a tuberculosis high-burden municipality in Northeast, Brazil[85]	2013	Government	National
86	Clusters of multidrug-resistant Mycobacterium tuberculosis cases, Europe[86]	2009	Government	Mixed
87	A genotypic and spatial epidemiologic analysis of Massachusetts' Mycobacterium tuberculosis cases from 2012 to 2015[87]	2018	na	na
88	The geno-spatio analysis of Mycobacterium tuberculosis complex in hot and cold spots of Guangxi, China[88]	2020	Government	National
89	Genotypic and Spatial Analysis of Mycobacterium tuberculosis Transmission in a High-Incidence Urban Setting[89]	2015	Government	National
90	Genotype heterogeneity of Mycobacterium tuberculosis within geospatial hotspots suggests foci of imported infection in Sydney, Australia[90]	2016	Non-profit organizations	National
91	Genomic epidemiology of tuberculosis in eastern Malaysia: insights for strengthening public health responses[91]	2021	Government, University	Mixed
92	Transmission and Drug Resistance Genotype of Multidrug-Resistant or Rifampicin-Resistant Mycobacterium tuberculosis in Chongqing, China[92]	2022	Government, non-profit	Mixed
93	Whole genome characterization, and geographical distribution of M. tuberculosis in central region of Veracruz, Mexico[93]	2022	Government	National
94	Transmission of multidrug-resistant tuberculosis in Beijing, China: An epidemiological and genomic analysis[94]	2022	Government	National
95	The role of prisons in disseminating tuberculosis in Brazil: A genomic epidemiology study[95]	2022	Government	Mixed
96	Genetic diversity of the Mycobacterium tuberculosis complex in San Luis Potosí, México[96]	2013	Government	National
97	Genetic diversity of Mycobacterium tuberculosis from Pará, Brazil, reveals a higher frequency of ancestral strains than previously reported in South America[97]	2017	Government	Mixed
98	Genetic diversity of drug and multidrug-resistant Mycobacterium tuberculosis circulating in Veracruz, Mexico[98]	2018	Government	National
99	Genetic diversity and distribution of Mycobacterium tuberculosis genotypes in Limpopo, South Africa[99]	2017	University	National
100	Genetic diversity and distribution dynamics of multidrug-resistant Mycobacterium tuberculosis isolates in Nepal[100]	2018	Government	International
101	Phylogeography and transmission of Mycobacterium tuberculosis spanning prisons and surrounding communities in Paraguay[101]	2023	Government	Mixed
102	Possible Outbreak of Streptomycin-Resistant Mycobacterium tuberculosis Beijing in Benin[102]	2009	Government, non-profit	Mixed
103	First Insight into the Molecular Epidemiology of Mycobacterium tuberculosis Isolates from the Minority Enclaves of Southwestern China[103]	2017	Government	National
104	A First Insight into the Genetic Diversity and Drug Susceptibility Pattern of Mycobacterium tuberculosis Complex in Zhejiang, China[104]	2016	Government	National
105	A field-validated approach using surveillance and genotyping data to estimate tuberculosis attributable to recent transmission in the United States[105]	2015	Government	National

106	Estimated rate of reactivation of latent tuberculosis infection in the United States, overall and by population subgroup[106]	2014	na	na
107	Distribution and identification of Mycobacterium tuberculosis lineage in Kashgar prefecture[107]	2022	Government	National
108	The Epidemiological Significance and Temporal Stability of Mycobacterial Interspersed Repetitive Units-Variable Number of Tandem Repeats-Based Method Applied to Mycobacterium tuberculosis in China[108]	2018	Government	Mixed
109	Epidemiological evaluation of spatiotemporal and genotypic clustering of Mycobacterium tuberculosis in Ontario, Canada[109]	2013	na	na
110	Spatial scale of tuberculosis transmission in Lima, Peru[110]	2022	Government	International
111	Dynamics of Mycobacterium tuberculosis Lineages in Oman, 2009 to 2018[111]	2022	Government	National
112	Mycobacterium tuberculosis ecology in Venezuela: epidemiologic correlates of common spoligotypes and a large clonal cluster defined by MIRU-VNTR-24[112]	2009	Government	National
113	Use of High-Resolution Geospatial and Genomic Data to Characterize Recent Tuberculosis Transmission, Botswana[113]	2023	Government	International
114	Distribution of molecular strains of Mycobacterium tuberculosis in an intermediate burden Asia Pacific city[114]	2021	Government	National
115	Genotypic and spatial analysis of transmission dynamics of tuberculosis in Shanghai, China: a 10-year prospective population-based surveillance study[115]	2023	Government	National
116	Distribution and Clonality of drug-resistant tuberculosis in South Africa[116]	2021	Government	International
117	Molecular epidemiology and drug resistance of widespread genotypes of Mycobacterium tuberculosis in northwestern Russia[117]	2009	na	na
118	Disease phenotypic and geospatial features vary across genetic lineages for Tuberculosis within Arkansas, 2010-2020[118]	2023	Government	National
119	Differences in the robustness of clusters involving the Mycobacterium tuberculosis strains most frequently isolated from immigrant cases in Madrid[119]	2010	Government	National
120	Detection of Tuberculosis Infection Hotspots Using Activity Spaces Based Spatial Approach in an Urban Tokyo, from 2003 to 2011[120]	2015	Government	National
121	Contextualizing tuberculosis risk in time and space: comparing time-restricted genotypic case clusters and geospatial clusters to evaluate the relative contribution of recent transmission to incidence of TB using nine years of case data from Michigan, USA[121]	2019	Government, University	National
122	Risk for Prison-to-Community Tuberculosis Transmission, Thailand, 2017-2020[122]	2023	Government	International
123	Combining molecular typing and spatial pattern analysis to identify areas of high tuberculosis transmission in a moderate-incidence county in Taiwan[123]	2017	Government	National
124	Clustering and recent transmission of Mycobacterium tuberculosis in a Chinese population[124]	2018	Government	National
125	Whole Genome Sequencing and Spatial Analysis Identifies Recent Tuberculosis Transmission Hotspots in Ghana[125]	2020	Non-profit organizations	International
126	Transmission, distribution and drug resistance-conferring mutations of extensively drug-resistant tuberculosis in the Western Cape Province, South Africa[126]	2022	Government, non-profit	Mixed
127	Association of Mycobacterium tuberculosis genotypes and clinical and epidemiological features - a multi-center study in Taiwan[127]	2012	Government	National
128	The Association between Mycobacterium Tuberculosis Genotype and Drug Resistance in Peru[128]	2015	Government, non-profit	International
129	Assessment of tuberculosis spatial hotspot areas in Antananarivo, Madagascar, by combining spatial analysis and genotyping[129]	2017	Government	National
130	Acquisition of second-line drug resistance and extensive drug resistance during recent transmission of Mycobacterium tuberculosis in rural China[130]	2015	Government	National

131	Accuracy of prospective space-time surveillance in detecting tuberculosis transmission[131]	2014	na	na
132	Genomic Sequencing Profiles of Mycobacterium tuberculosis in Mandalay Region, Myanmar[132]	2023	Government	International
133	Transmission dynamics and phylogeography of Mycobacterium tuberculosis in China based on whole-genome phylogenetic analysis[133]	2024	Government	National
134	Population structure of Mycobacterium tuberculosis in El Oro: A first insight into Ecuador-Peru tuberculosis transmission[134]	2024	na	na
135	Prison as a driver of recent transmissions of multidrug-resistant tuberculosis in Callao, Peru: a cross-sectional study[135]	2024	Government	International
136	Strain structure analysis of Mycobacterium tuberculosis circulating among HIV negative, positive and drug resistant TB patients attending chest clinics in Western Kenya[136]	2023	Government	Mixed
137	Utility of Mycobacterium tuberculosis Genome Sequencing Snapshots to Assess Transmission Dynamics Over Time[137]	2024	Government	International
138	Whole Genome Sequence-Based Analyses of Drug Resistance Characteristics, Genetic Diversity, and Transmission Dynamics of Drug-Resistant Mycobacterium tuberculosis in Urumqi City[138]	2024	Government	National
139	Transmission dynamics of drug-resistant tuberculosis in Ningbo, China: an epidemiological and genomic analysis[139]	2024	Government	National
140	Identifying local foci of tuberculosis transmission in Moldova using a spatial multinomial logistic regression model[140]	2024	Government	International
141	Genomic and spatial analysis reveal the transmission dynamics of tuberculosis in areas with high incidence of Zhejiang, China: A prospective cohort study[141]	2024	Government	National
142	Determining the risk-factors for molecular clustering of drug-resistant tuberculosis in South Africa[142]	2023	University	National

Table S2. Included studies by characteristics and question types.

Article ID	**Country**	**Incidence (20;40;50)**	**DR (MDR/XDR)**	**Number of total TB cases**	**Spatial type**	**Aggregation level**	**Duration (year)**	**Genotyping methods**	**Genomic analysis methods**	**Question types**
1	Greenland	high	na	272	Aggregated	second	1	RFLP	Genotype	a
2	India	high	na	68	Aggregated	first	na	RFLP	Genotype	a
3	US	low	na	1324	Aggregated	first	2;6	RFLP	Genotype	a
4	Brazil	high	na	1991	Aggregated	first	9	spoligotyping	Genotype	a
5	Argentina, Brazil, Chile, Colombia, Venezuela and Spain	na	MDR	1078	Aggregated	Nations	5	spoligotyping; RFLP	Genotype	a
6	Mexico	na	na	414	Aggregated	first	12	spoligotyping	Genotype	a
7	India	high	DR	628	Aggregated	first	4	spoligotyping	Genotype	a
8	Mexico	high	na	140	Aggregated	second	3	MIRU	Genotype	a
9	Chile	low; mid	na	458	Aggregated	second	2	spoligotyping	Genotype	a
10	Zimbabwe	high	na	141	Point	na	1	spoligotyping	Genotype	a
11	South Africa	high	XDR	344	Point; Aggregated	second	4	WGS	WGS	a;d
12	Brazil	mid	na	151	Point	na	3	WGS	WGS	a;d
13	US	na	na	724	Aggregated	second	2	RFLP; Other	Genotype	a
14	Malaysia	high	na	439	Aggregated	first	2	spoligotyping; RFLP	Genotype	a
15	US	low	DR	201	Aggregated	second	3	RFLP	Genotype	a
16	Colombia	high	DR	53	Aggregated	first	2	RFLP	Genotype	a
17	US and Mexico	na	DR	919	Aggregated	first	9	spoligotyping; RFLP	Genotype	a
18	US	na	na	1764	Point	na	5	spoligotyping; MIRU; RFLP	Genotype	a
19	South Africa	high	na	780	Point	na	6	spoligotyping; RFLP	Genotype	a;b
20	Japan	low	MDR/XDR	55	Aggregated	second	1	spoligotyping; MIRU; RFLP	Genotype	a
21	US	low	DR	54	Aggregated	second	7	spoligotyping; MIRU; RFLP	Genotype	a

22	Uganda	high	na	54	Point	na	1	spoligotyping	Genotype	a
23	Uganda	high	na	84/131	Point	na	3	spoligotyping; MIRU	Genotype	a
24	Portugal	mid	na	144	Point	na	2	MIRU	Genotype	a;d
25	Mali	high	na	245	Aggregated	second	3	spoligotyping; MIRU	Genotype	a
26	South Africa	high	DR	1157	Aggregated	second	3	spoligotyping	Genotype	a
27	Canada	low	na	629	Aggregated	second	8	RFLP	Genotype	a
28	Denmark	low	na	3936	Aggregated	first	10	RFLP	Genotype	a
29	US	low	na	1384	Aggregated	second	7	spoligotyping; MIRU	Genotype	a;b;c
30	Mexico	mid	na	267	Point	na	2	spoligotyping	Genotype	a;b;c
31	US	low	na	519	Aggregated	second	5	spoligotyping; MIRU	Genotype	a;b;c
32	Bangladesh	high	MDR	544	Point	na	7	spoligotyping; MIRU	Genotype	a
33	Taiwan	na	na	969	Point; Aggregated	second	3	spoligotyping; MIRU	Genotype	a;b
34	China	high	na	1620	Point; Aggregated	second	7	WGS	WGS	a;b;d
35	US	low	na	na	Aggregated	second	5	spoligotyping; MIRU	Genotype	a;b
36	US	low	na	na	Aggregated	second	7	spoligotyping; MIRU	Genotype	a;b
37	US	low	na	36860	Aggregated	second	5	spoligotyping; MIRU	Genotype	a;b;c
38	Zimbabwe	high	na	516	Point; Aggregated	second	1	spoligotyping; MIRU	Genotype	a
39	Canada	low	na	20/1823	Point	na	12	spoligotyping; RFLP	Genotype	a
40	China	high	DR	223	Aggregated	second	2	spoligotyping; RFLP	Genotype	a
41	China	na	MDR	299	Point	na	4	MIRU	Genotype	a;b
42	Australia	low	na	930	Aggregated	second	3	spoligotyping; MIRU	Genotype	a
43	China	na	na	2346	Aggregated	first	3	spoligotyping	Genotype	a
44	Poland	na	MDR	46	Aggregated	first	1	spoligotyping	Genotype	a
45	US	low	na	2531	Aggregated	second	5	spoligotyping; MIRU	Genotype	a;b

46	Ghana	na	na	2551	Point; Aggregated	second	3	spoligotyping; Other	Genotype	a;b
47	United States	low	na	1655	Aggregated	second	7	spoligotyping; MIRU	Genotype	a;b
48	Moldova	high	na	2182	Aggregated	first	2	WGS	WGS	a;d
49	Ethiopia	high	na	72	Aggregated	second	1	MIRU	Genotype	a;b
50	China	high	MDR	417	Point; Aggregated	second	5	WGS	WGS	a;b
51	Hong Kong	high	na	2332	Aggregated	second	3	RFLP	Genotype	a
52	Myanmar	high	DR	109	Point; Aggregated	second	1	WGS	WGS	a
53	Taiwan	high	na	421	Aggregated	first	1	spoligotyping	Genotype	a
54	US	low	na	576	Aggregated	second	6	spoligotyping; MIRU	Genotype	a;b
55	China	high	na	975	Aggregated	first	1	MIRU	Genotype	a
56	Botswana	high	na	1796	Point	na	5	MIRU	Genotype	a;b;c
57	Peru	na	MDR	35	Point	na	2	WGS	WGS	a;b;c; d
58	Oman	low	na	70	Point	na	1	WGS	WGS	a
59	South Korea	na	na	80	Aggregated	first	1	spoligotyping; MIRU; RFLP	Genotype	a
60	Botswana	high	na	1924	Aggregated	second	5	MIRU	Genotype	a
61	Canada	low	na	35	Point	second	14	MIRU; RFLP	Genotype	a;b
62	Uganda	high	DR	973	Aggregated	first	1	RFLP; Other	Genotype	a
63	Guadeloupe, Martinique, and French Guiana	low; high	na	744	Aggregated	Nations	10	spoligotyping; Other	Genotype	a
64	Nigeria	high	na	549	Aggregated	first	2	spoligotyping	Genotype	a
65	Mozambique	na	DR	543	Aggregated	first	2	spoligotyping; MIRU; RFLP	Genotype	a
66	Ghana	na	na	613	Aggregated	first	5	spoligotyping; MIRU	Genotype	a
67	Botswana	high	na	24	Point	na	4	MIRU	Genotype	a
68	Czech Republic	low	na	155	Aggregated	first	1	spoligotyping; MIRU; RFLP	Genotype	a
69	Cambodia	high	na	161	Aggregated	first	5	spoligotyping; MIRU	Genotype	a;b;c; d
70	Ethiopia	na	na	230	Aggregated	second	1	spoligotyping	Genotype	a

71	Taiwan	high	na	171	Aggregated	first	6	spoligotyping; MIRU	Genotype	a
72	Canada	low	na	706	Point; Aggregated	second	5	spoligotyping; RFLP	Genotype	a;b;c
73	Canada	high	na	228	Aggregated	second	19	RFLP	Genotype	a
74	China	high	na	1586	Aggregated	first	3	MIRU	Genotype	a
75	Brazil	high	na	342/717	Point	na	3	spoligotyping; RFLP	Genotype	a
76	Peru	high	MDR	3286	Point	na	4	MIRU	Genotype	a;b
77	United Kingdom	low	na	1484	Aggregated	second	3	MIRU	Genotype	a;b
78	US	low	na	538	Aggregated	second	10	spoligotyping; RFLP	Genotype	a
79	Thailand	na	MDR	2071	Aggregated	first	4	WGS	WGS	a
80	Argentina	na	MDR/XDR	787	Aggregated	second	7	spoligotyping; RFLP	Genotype	a
81	Canada	low	DR	2819	Point	na	2	RFLP	Genotype	a;b
82	South Africa	high	XDR	699	Aggregated	first	2	spoligotyping	Genotype	a
83	Nepal	high	MDR	145	Aggregated	second	6	spoligotyping; MIRU	Genotype	a
84	Mexico	mid	na	1370	Point	na	6	spoligotyping; RFLP	Genotype	a;b
85	Brazil	high	na	115	Point	na	2	spoligotyping	Genotype	a
86	All 53 countries of the WHO European Region	na	MDR	2494	Aggregated	Nations	5	spoligotyping; MIRU; RFLP	Genotype	a
87	US	low	na	543	Aggregated	second	4	spoligotyping; MIRU	Genotype	a
88	China	high	na	291	Aggregated	second	1	WGS	WGS	a;d
89	Brazil	high	na	503	Point; Aggregated	second	5	spoligotyping; RFLP	Genotype	a;b
90	Australia	low to high	na	1692	Aggregated	second	5	MIRU	Genotype	a;b
91	Malaysia	high	na	216	Aggregated	second	2	WGS	WGS	a
92	China	high	MDR	200	Point	na	4	WGS	WGS	a;d
93	Mexico	mid	na	25	Point	na	2	WGS	WGS	a
94	China	low	MDR	241	Point	na	3	WGS	WGS	a;b
95	Brazil	mid	na	935	Aggregated	second	6	WGS	WGS	a

96	Mexico	low	na	237	Aggregated	second	5	spoligotyping; MIRU	Genotype	a
97	Brazil	mid	na	980	Point	na	4	spoligotyping; MIRU	Genotype	a
98	Mexico	mid	MDR	112	Aggregated	second	2	spoligotyping; MIRU	Genotype	a
99	South Africa	high	na	215	Aggregated	second	1	spoligotyping	Genotype	a
100	Nepal	high	MDR	498	Aggregated	first	5	spoligotyping; MIRU; Other	Genotype	a
101	Paraguay	mid	na	471	Aggregated	second	6	WGS	WGS	a
102	Benin	na	DR	194	Point	na	2	spoligotyping; MIRU	Genotype	a
103	China	high	na	271	Aggregated	second	1	spoligotyping; MIRU	Genotype	a
104	China	high	na	940	Aggregated	second	1	spoligotyping	Genotype	a
105	US	low	na	2935	Aggregated	second	3	RFLP	Genotype	a;b
106	US	low	na	31841	Aggregated	second	3	spoligotyping; MIRU	Genotype	a;b
107	China	high	na	161	Aggregated	second	2	WGS	WGS	a
108	China	high	na	982	point	na	4	spoligotyping; MIRU; RFLP	Genotype	a
109	Canada	low	na	2186	Aggregated	second	5	spoligotyping; MIRU	Genotype	a;b;c
110	Peru	high	na	2440	Aggregated	second	4	WGS	WGS	a;b;d
111	Oman	low	na	2539	Aggregated	first	10	spoligotyping; MIRU	Genotype	a
112	Venezuela	mid	na	1298	Aggregated	first	10	spoligotyping; MIRU	Genotype	a
113	Botswana	high	na	1449	Point	na	5	WGS	WGS	a;b;d
114	Hong Kong	high	na	382	Point; Aggregated	second	2	MIRU	Genotype	a
115	China	high	na	2212	Point	na	10	WGS	WGS	a;b;d
116	South Africa	high	DR	3007	Aggregated	first	5	spoligotyping; MIRU	Genotype	a
117	Russia	na	DR	192	Aggregated	first	3	spoligotyping; RFLP	Genotype	a
118	US	low	na	686	Aggregated	second	11	spoligotyping; MIRU	Genotype	a;b
119	Spain	low	na	1507	Point	second	3;4	spoligotyping; MIRU; RFLP	Genotype	a

120	Japan	low; mid	na	643	Point; Aggregated	second	9	spoligotyping; RFLP	Genotype	a;b;c
121	US	low	na	1235	Point; Aggregated	second	9	spoligotyping; MIRU	Genotype	a;b
122	Thailand	na	na	532+60	Point	na	3	WGS	WGS	a;b
123	Taiwan	high	na	303	Point	second	1	spoligotyping; MIRU	Genotype	a;b
124	China	high	na	912	Aggregated	second	2	MIRU	Genotype	a
125	Ghana	na	na	452	Point	na	4	WGS	WGS	a
126	South Africa	high	XDR	461	Aggregated	second	12	WGS	WGS	a
127	Taiwan	high	na	516	Aggregated	first	3	spoligotyping; MIRU	Genotype	a
128	Peru	na	DR	2086	Aggregated	second	3	spoligotyping; MIRU	Genotype	a
129	Madagascar	high	na	467	Aggregated	second	1	spoligotyping	Genotype	a;b
130	China	high	XDR	1332	Aggregated	first	3	spoligotyping; MIRU	Genotype	a
131	Canada	na	na	1566	Aggregated	second	10	spoligotyping; RFLP	Genotype	a;b
132	Myanmar	mid	na	151	Aggregated	second	1	WGS	WGS	a
133	China	high	na	3204	Aggregated	first	10	WGS	WGS	a
134	Ecuador	high	na	56	Aggregated	first	5	spoligotyping; MIRU	Genotype	a
135	Peru	high	MDR	171	Point	na	2	WGS	WGS	a;b
136	Kenya	high	DR	553	Aggregated	first	2	spoligotyping	Genotype	a
137	Peru	high	na	151	Point	na	2	WGS	WGS	a;d
138	China	high	DR	207	Point	na	6	WGS	WGS	a;b
139	China	high	DR	130	Point	na	3	WGS	WGS	a;b;d
140	Moldova	high	na	2236	Aggregated	second	2	WGS	WGS	a;b;c
141	China	high	na	532	Point	na	6	WGS	WGS	a;b;d
142	South Africa	high	DR	2893	Aggregated	second	4	spoligotyping; MIRU	Genotype	a

References of all included studies:

1. Yang Z, De Haas P, Van Soolingen D, Van Embden J, Andersen AB. Restriction fragment length polymorphism Mycobacterium tuberculosis strains isolated from Greenland during 1992: evidence of tuberculosis transmission between Greenland and Denmark. *Journal of clinical microbiology* 1994; **32**(12): 3018-25.
2. Narayanan S, Sahadevan R, Narayanan P, Krishnamurthy P, Paramasivan C, Prabhakar R. Restriction fragment length polymorphism of Mycobacterium tuberculosis strains from various regions of India, using direct repeat probe. *Indian Journal of Medical Research* 1997; **106**(Nov): 447-54.
3. Yang Z, Barnes PF, Chaves F, Eisenach KD, Weis SE, Bates JH, Cave MD. Diversity of DNA fingerprints of Mycobacterium tuberculosis isolates in the United States. *Journal of clinical microbiology* 1998; **36**(4): 1003-7.
4. Gomes HM, Elias AR, Oelemann MAC, et al. Spoligotypes of Mycobacterium tuberculosis complex isolates from patients residents of 11 states of Brazil. *Infection, Genetics and Evolution* 2012; **12**(4): 649-56.
5. Ritacco V, Iglesias M-J, Ferrazoli L, et al. Conspicuous multidrug-resistant Mycobacterium tuberculosis cluster strains do not trespass country borders in Latin America and Spain. *Infection, Genetics and Evolution* 2012; **12**(4): 711-7.
6. Vera-Cabrera L, Ramos-Alvarez J, Molina-Torres CA, Rivera-Morales LG, Rendón A, Quiñones-Falconi F, Ocampo-Candiani J. Comparative Mycobacterium tuberculosis spoligotype distribution in Mexico. *Journal of clinical microbiology* 2014; **52**(8): 3049-52.
7. Singh J, Sankar MM, Kumar P, et al. Genetic diversity and drug susceptibility profile of Mycobacterium tuberculosis isolated from different regions of India. *Journal of Infection* 2015; **71**(2): 207-19.
8. Flores-López CA, Zenteno-Cuevas R, Laniado-Laborín R, et al. Molecular epidemiology of Mycobacterium tuberculosis in Baja California, Mexico: A result of human migration? *Infection, Genetics and Evolution* 2017; **55**: 378-83.
9. Lagos J, Couvin D, Arata L, et al. Analysis of Mycobacterium tuberculosis genotypic lineage distribution in Chile and neighboring countries. *PLoS One* 2016; **11**(8): e0160434.
10. Heyderman R, Goyal M, Roberts P, et al. Pulmonary tuberculosis in Harare, Zimbabwe: analysis by spoligotyping. *Thorax* 1998; **53**(5): 346-50.
11. Nelson KN, Shah NS, Mathema B, et al. Spatial patterns of extensively drug-resistant tuberculosis transmission in KwaZulu-Natal, South Africa. *The Journal of infectious diseases* 2018; **218**(12): 1964-73.
12. Verza M, Scheffer MC, Salvato RS, et al. Genomic epidemiology of Mycobacterium tuberculosis in Santa catarina, Southern Brazil. *Scientific reports* 2020; **10**(1): 12891.
13. Bradford WZ, Koehler J, El-Hajj H, et al. Dissemination of Mycobacterium tuberculosis across the San Francisco Bay area. *Journal of Infectious Diseases* 1998; **177**(4): 1104-7.
14. Dale JW, Nor RM, Ramayah S, Tang TH, Zainuddin ZF. Molecular epidemiology of tuberculosis in Malaysia. *Journal of Clinical Microbiology* 1999; **37**(5): 1265-8.

15. Wilson RW, Yang Z, Kelley M, et al. Evidence from molecular fingerprinting of limited spread of drug-resistant tuberculosis in Texas. *Journal of clinical microbiology* 1999; **37**(10): 3255-9.
16. Gómez-Marin JE, León Franco CI, Inirida Guerrero M, Rigouts L, Portaels F. IS6110 fingerprinting of sensitive and resistant strains (1991-1992) of Mycobacterium tuberculosis in Colombia. *Memórias do Instituto Oswaldo Cruz* 2002; **97**: 1005-8.
17. Quitugua TN, Seaworth BJ, Weis SE, et al. Transmission of drug-resistant tuberculosis in Texas and Mexico. *Journal of Clinical Microbiology* 2002; **40**(8): 2716-24.
18. Mathema B, Bifani PJ, Driscoll J, et al. Identification and evolution of an IS 6110 low-copy-number Mycobacterium tuberculosis cluster. *The Journal of infectious diseases* 2002; **185**(5): 641-9.
19. Richardson M, van Lill SW P, van der Spuy G D, et al. Historic and recent events contribute to the disease dynamics of Beijing-like Mycobacterium tuberculosis isolates in a high incidence region. *The International Journal of Tuberculosis and Lung Disease* 2002; **6**(11): 1001-11.
20. Murase Y, Maeda S, Yamada H, et al. Clonal expansion of multidrug-resistant and extensively drug-resistant tuberculosis, Japan. *Emerging infectious diseases* 2010; **16**(6): 948.
21. Perri BR, Proops D, Moonan PK, et al. Mycobacterium tuberculosis cluster with developing drug resistance, New York, New York, USA, 2003–2009. *Emerging infectious diseases* 2011; **17**(3): 372.
22. Chamie G, Wandera B, Marquez C, Kato-Maeda M, Kamya MR, Havlir DV, Charlebois ED. Identifying locations of recent TB transmission in rural Uganda: a multidisciplinary approach. *Tropical Medicine & International Health* 2015; **20**(4): 537-45.
23. Chamie G, Kato-Maeda M, Emperador DM, et al. Spatial overlap links seemingly unconnected genotype-matched TB cases in rural Uganda. *PLoS One* 2018; **13**(2): e0192666.
24. Rito T, Matos C, Carvalho C, et al. A complex scenario of tuberculosis transmission is revealed through genetic and epidemiological surveys in Porto. *BMC Infectious Diseases* 2018; **18**: 1-12.
25. Kone B, Somboro AM, Kone M, et al. Molecular epidemiology and genetic diversity of Mycobacterium tuberculosis complex in referral health centers of Bamako, Mali: What is new? *International Journal of Infectious Diseases* 2022; **117**: 204-11.
26. Faye LM, Hosu MC, Vasaikar S, Dippenaar A, Oostvogels S, Warren RM, Apalata T. Spatial distribution of drug-resistant mycobacterium tuberculosis infections in rural Eastern Cape province of South Africa. *Pathogens* 2023; **12**(3): 475.
27. Blackwood KS, Al-Azem A, Elliott LJ, Hershfield ES, Kabani AM. Conventional and molecular epidemiology of tuberculosis in Manitoba. *BMC infectious diseases* 2003; **3**: 1-11.
28. Lillebaek T, Dirksen A, Kok-Jensen A, Andersen Å. A dominant Mycobacterium tuberculosis strain emerging in Denmark. *The International Journal of Tuberculosis and Lung Disease* 2004; **8**(8): 1001-6.
29. Prussing C, Castillo-Salgado C, Baruch N, Cronin WA. Geo-epidemiologic and molecular characterization to identify social, cultural, and economic factors where targeted tuberculosis control activities can reduce incidence in Maryland, 2004–2010. *Public health reports* 2013; **128**(6_suppl3): 104-14.

30. Nava-Aguilera E, Lopez-Vidal Y, Harris E, et al. Clustering of Mycobacterium tuberculosis cases in Acapulco: Spoligotyping and risk factors. *Journal of Immunology Research* 2011; **2011**(1): 408375.
31. Oren E, Narita M, Nolan C, Mayer J. Neighborhood socioeconomic position and tuberculosis transmission: a retrospective cohort study. *BMC infectious diseases* 2014; **14**: 1-11.
32. Rahman SM, Rahman A, Nasrin R, et al. Molecular epidemiology and genetic diversity of multidrug-resistant Mycobacterium tuberculosis isolates in Bangladesh. *Microbiology spectrum* 2022; **10**(1): e01848-21.
33. Ng I-C, Wen T-H, Yang S-T, Fang C-T, Hsueh P-R. Detecting tuberculosis clusters in urban neighborhoods, Taipei, Taiwan: Linking geographic and genotyping evidence. *Applied Geography* 2019; **104**: 56-64.
34. Yang C, Lu L, Warren JL, et al. Internal migration and transmission dynamics of tuberculosis in Shanghai, China: an epidemiological, spatial, genomic analysis. *The Lancet Infectious Diseases* 2018; **18**(7): 788-95.
35. Kammerer JS, Shang N, Althomsons SP, Haddad MB, Grant J, Navin TR. Using statistical methods and genotyping to detect tuberculosis outbreaks. *International journal of health geographics* 2013; **12**: 1-8.
36. Althomsons SP, Kammerer JS, Shang N, Navin TR. Using routinely reported tuberculosis genotyping and surveillance data to predict tuberculosis outbreaks. *PLoS One* 2012; **7**(11): e48754.
37. Moonan PK, Ghosh S, Oeltmann JE, Kammerer JS, Cowan LS, Navin TR. Using genotyping and geospatial scanning to estimate recent Mycobacterium tuberculosis transmission, United States. *Emerging infectious diseases* 2012; **18**(3): 458.
38. Easterbrook PJ, Gibson A, Murad S, et al. High rates of clustering of strains causing tuberculosis in Harare, Zimbabwe: a molecular epidemiological study. *Journal of Clinical Microbiology* 2004; **42**(10): 4536-44.
39. Tan de Bibiana J, Rossi C, Rivest P, et al. Tuberculosis and homelessness in Montreal: a retrospective cohort study. *BMC Public Health* 2011; **11**: 1-10.
40. Hu Y, Mathema B, Jiang W, Kreiswirth B, Wang W, Xu B. Transmission pattern of drug-resistant tuberculosis and its implication for tuberculosis control in eastern rural China. *PLoS One* 2011; **6**(5): e19548.
41. Ge E, Li D, Luo M, Tsui KWS, Waye MMY, Shen X, Wei X. Transmission of multidrug-resistant tuberculosis in Shanghai: roles of residential status. *Int J Tuberc Lung Dis* 2018; **22**(12): 1462-8.
42. Gallego B, Sintchenko V, Jelfs P, Coiera E, Gilbert GL. Three-year longitudinal study of genotypes of Mycobacterium tuberculosis in a low prevalence population. *Pathology* 2010; **42**(3): 267-72.
43. Dong H, Liu Z, Lv B, et al. Spoligotypes of Mycobacterium tuberculosis from different Provinces of China. *J Clin Microbiol* 2010; **48**(11): 4102-6.
44. Jagielski T, Augustynowicz-Kopec E, Zozio T, Rastogi N, Zwolska Z. Spoligotype-based comparative population structure analysis of multidrug-resistant and isoniazid-monoresistant Mycobacterium tuberculosis complex clinical isolates in Poland. *J Clin Microbiol* 2010; **48**(11): 3899-909.
45. Séraphin MN, Lauzardo M, Doggett RT, Zabala J, Morris JG, Jr., Blackburn JK. Spatiotemporal Clustering of Mycobacterium tuberculosis Complex Genotypes in Florida: Genetic Diversity Segregated by Country of Birth. *PLoS One* 2016; **11**(4): e0153575.

46. Yeboah-Manu D, Asare P, Asante-Poku A, et al. Spatio-Temporal Distribution of Mycobacterium tuberculosis Complex Strains in Ghana. *PLoS One* 2016; **11**(8): e0161892.
47. Agarwal S, Nguyen DT, Teeter LD, Graviss EA. Spatial-temporal distribution of genotyped tuberculosis cases in a county with active transmission. *BMC Infect Dis* 2017; **17**(1): 378.
48. Yang C, Sobkowiak B, Naidu V, et al. Phylogeography and transmission of M. tuberculosis in Moldova: A prospective genomic analysis. *PLoS Med* 2022; **19**(2): e1003933.
49. Tafess K, Beyen TK, Girma S, Girma A, Siu G. Spatial clustering and genetic diversity of Mycobacterium tuberculosis isolate among pulmonary tuberculosis suspected patients, Arsi Zone, Ethiopia. *BMC Pulm Med* 2021; **21**(1): 206.
50. Jiang Q, Liu Q, Ji L, et al. Citywide Transmission of Multidrug-resistant Tuberculosis Under China's Rapid Urbanization: A Retrospective Population-based Genomic Spatial Epidemiological Study. *Clin Infect Dis* 2020; **71**(1): 142-51.
51. Chan-Yeung M, Yeh AGO, Tam CM, Kam KM, Leung CC, Yew WW, Lam CW. Socio-demographic and geographic indicators and distribution of tuberculosis in Hong Kong: A spatial analysis. *International Journal of Tuberculosis and Lung Disease* 2005; **9**(12): 1320-6.
52. Maung HMW, Palittapongarnpim P, Aung HL, Surachat K, Nyunt WW, Chongsuvivatwong V. Geno-Spatial Distribution of Mycobacterium Tuberculosis and Drug Resistance Profiles in Myanmar-Thai Border Area. *Trop Med Infect Dis* 2020; **5**(4).
53. Jou R, Chiang CY, Huang WL. Distribution of the Beijing family genotypes of Mycobacterium tuberculosis in Taiwan. *J Clin Microbiol* 2005; **43**(1): 95-100.
54. Lindquist S, Allen S, Field K, Ghosh S, Haddad MB, Narita M, Oren E. Prioritizing tuberculosis clusters by genotype for public health action, Washington, USA. *Emerg Infect Dis* 2013; **19**(3): 493-6.
55. Ni X, Zhu C, Li Q, et al. Epidemiology characteristics of the clonal complexes of Mycobacterium tuberculosis Lineage 4 in China. *Infect Genet Evol* 2020; **84**: 104363.
56. Zetola NM, Moonan PK, Click E, et al. Population-Based Geospatial and Molecular Epidemiologic Study of Tuberculosis Transmission Dynamics, Botswana, 2012-2016. *Emerg Infect Dis* 2021; **27**(3): 835-44.
57. Bui DP, Chandran SS, Oren E, Brown HE, Harris RB, Knight GM, Grandjean L. Community transmission of multidrug-resistant tuberculosis is associated with activity space overlap in Lima, Peru. *BMC Infect Dis* 2021; **21**(1): 275.
58. Al-Jardani A, Al Yaquobi F, Adikaram C, et al. Genomic and geospatial epidemiology of Mycobacterium tuberculosis in Oman: first national insight using whole genome sequencing. *Int J Infect Dis* 2023; **130 Suppl 1**: S4-s11.
59. Kang HY, Wada T, Iwamoto T, et al. Phylogeographical particularity of the Mycobacterium tuberculosis Beijing family in South Korea based on international comparison with surrounding countries. *J Med Microbiol* 2010; **59**(Pt 10): 1191-7.
60. Click ES, Finlay A, Oeltmann JE, et al. Phylogenetic diversity of Mycobacterium tuberculosis in two geographically distinct locations in Botswana - The Kopanyo Study. *Infect Genet Evol* 2020; **81**: 104232.
61. Aho J, Lacroix C, Bazargani M, et al. Outbreak of tuberculosis among substance users and homeless people in Greater Montréal, Canada, 2003-2016. *Can Commun Dis Rep* 2017; **43**(3-4): 72-6.

62. Ezati N, Lukoye D, Wampande EM, et al. The Mycobacterium tuberculosis Uganda II family and resistance to first-line anti-tuberculosis drugs in Uganda. *BMC Infect Dis* 2014; **14**: 703.
63. Brudey K, Filliol I, Ferdinand S, et al. Long-term population-based genotyping study of Mycobacterium tuberculosis complex isolates in the French departments of the Americas. *J Clin Microbiol* 2006; **44**(1): 183-91.
64. Molina-Moya B, Gomgnimbou MK, Spinasse L, et al. Mycobacterium tuberculosis complex genotypes circulating in Nigeria based on spoligotyping obtained from Ziehl-Neelsen stained slides extracted DNA. *PLoS Negl Trop Dis* 2018; **12**(2): e0006242.
65. Viegas SO, Machado A, Groenheit R, et al. Mycobacterium tuberculosis Beijing genotype is associated with HIV infection in Mozambique. *PLoS One* 2013; **8**(8): e71999.
66. Asante-Poku A, Yeboah-Manu D, Otchere ID, et al. Mycobacterium africanum is associated with patient ethnicity in Ghana. *PLoS Negl Trop Dis* 2015; **9**(1): e3370.
67. Surie D, Fane O, Finlay A, et al. Molecular, Spatial, and Field Epidemiology Suggesting TB Transmission in Community, Not Hospital, Gaborone, Botswana. *Emerg Infect Dis* 2017; **23**(3): 487-90.
68. Prodinger WM, Polanecký V, Kozáková B, et al. Molecular epidemiology of tuberculosis in the Czech Republic, 2004: analysis of M. tuberculosis complex isolates originating from the city of prague, south Moravia and the Moravian-Silesian region. *Cent Eur J Public Health* 2006; **14**(4): 168-74.
69. Schopfer K, Rieder HL, Steinlin-Schopfer JF, et al. Molecular epidemiology of tuberculosis in Cambodian children. *Epidemiol Infect* 2015; **143**(5): 910-21.
70. Merid Y, Hailu E, Habtamu G, et al. Molecular Epidemiology of Mycobacterium tuberculosis strains isolated from pulmonary tuberculosis patients in south Ethiopia. *J Infect Dev Ctries* 2021; **15**(9): 1299-307.
71. Chen YY, Chang JR, Huang WF, et al. Molecular epidemiology of Mycobacterium tuberculosis in aboriginal peoples of Taiwan, 2006-2011. *J Infect* 2014; **68**(4): 332-7.
72. Haase I, Olson S, Behr MA, et al. Use of geographic and genotyping tools to characterise tuberculosis transmission in Montreal. *Int J Tuberc Lung Dis* 2007; **11**(6): 632-8.
73. Pepperell C, Chang AH, Wobeser W, Parsonnet J, Hoeppner VH. Local epidemic history as a predictor of tuberculosis incidence in Saskatchewan Aboriginal communities. *Int J Tuberc Lung Dis* 2011; **15**(7): 899-905.
74. Wan K, Liu J, Hauck Y, et al. Investigation on Mycobacterium tuberculosis diversity in China and the origin of the Beijing clade. *PLoS One* 2011; **6**(12): e29190.
75. Pinho STR, Pereira SM, Miranda JGV, et al. Investigating extradomiciliary transmission of tuberculosis: An exploratory approach using social network patterns of TB cases and controls and the genotyping of Mycobacterium tuberculosis. *Tuberculosis (Edinb)* 2020; **125**: 102010.
76. Zelner JL, Murray MB, Becerra MC, et al. Identifying Hotspots of Multidrug-Resistant Tuberculosis Transmission Using Spatial and Molecular Genetic Data. *J Infect Dis* 2016; **213**(2): 287-94.

77. Saavedra-Campos M, Welfare W, Cleary P, et al. Identifying areas and risk groups with localised Mycobacterium tuberculosis transmission in northern England from 2010 to 2012: spatiotemporal analysis incorporating highly discriminatory genotyping data. *Thorax* 2016; **71**(8): 742-8.
78. Oppong JR, Denton CJ, Moonan PK, Weis SE. Foreign-Born Status and Geographic Patterns of Tuberculosis Genotypes in Tarrant County, Texas. *Prof Geogr* 2007; **59**(4): 478-91.
79. Nonghanphithak D, Chaiprasert A, Smithtikarn S, et al. Clusters of drug-resistant mycobacterium tuberculosis detected by whole-genome sequence analysis of nationwide sample, Thailand, 2014–2017. *Emerg Infect Dis* 2021; **27**(3): 813-22.
80. Ritacco V, López B, Ambroggi M, et al. HIV infection and geographically bound transmission of drug-resistant tuberculosis, Argentina. *Emerg Infect Dis* 2012; **18**(11): 1802-10.
81. Brassard P, Henry KA, Schwartzman K, Jomphe M, Olson SH. Geography and genealogy of the human host harbouring a distinctive drug-resistant strain of tuberculosis. *INFECTION GENETICS AND EVOLUTION* 2008; **8**(3): 247-57.
82. Mlambo CK, Warren RM, Poswa X, Victor TC, Duse AG, Marais E. Genotypic diversity of extensively drug-resistant tuberculosis (XDR-TB) in South Africa. *Int J Tuberc Lung Dis* 2008; **12**(1): 99-104.
83. Shah Y, Maharjan B, Thapa J, et al. High diversity of multidrug-resistant Mycobacterium tuberculosis Central Asian Strain isolates in Nepal. *Int J Infect Dis* 2017; **63**: 13-20.
84. Blanco-Guillot F, Castañeda-Cediel ML, Cruz-Hervert P, et al. Genotyping and spatial analysis of pulmonary tuberculosis and diabetes cases in the state of Veracruz, Mexico. *PLoS One* 2018; **13**(3): e0193911.
85. Luiz Rdos S, Suffys P, Barroso EC, et al. Genotyping and drug resistance patterns of Mycobacterium tuberculosis strains observed in a tuberculosis high-burden municipality in Northeast, Brazil. *Braz J Infect Dis* 2013; **17**(3): 338-45.
86. Devaux I, Kremer K, Heersma H, Van Soolingen D. Clusters of multidrug-resistant Mycobacterium tuberculosis cases, Europe. *Emerg Infect Dis* 2009; **15**(7): 1052-60.
87. Vindenes T, Jordan MR, Tibbs A, Stopka TJ, Johnson D, Cochran J. A genotypic and spatial epidemiologic analysis of Massachusetts' Mycobacterium tuberculosis cases from 2012 to 2015. *Tuberculosis (Edinb)* 2018; **112**: 20-6.
88. Lin D, Cui Z, Chongsuvivatwong V, et al. The geno-spatio analysis of Mycobacterium tuberculosis complex in hot and cold spots of Guangxi, China. *BMC Infect Dis* 2020; **20**(1): 462.
89. Ribeiro FK, Pan W, Bertolde A, et al. Genotypic and Spatial Analysis of Mycobacterium tuberculosis Transmission in a High-Incidence Urban Setting. *Clin Infect Dis* 2015; **61**(5): 758-66.
90. Gurjav U, Jelfs P, Hill-Cawthorne GA, Marais BJ, Sintchenko V. Genotype heterogeneity of Mycobacterium tuberculosis within geospatial hotspots suggests foci of imported infection in Sydney, Australia. *Infect Genet Evol* 2016; **40**: 346-51.
91. Bainomugisa A, Meumann EM, Rajahram GS, et al. Genomic epidemiology of tuberculosis in eastern Malaysia: insights for strengthening public health responses. *Microb Genom* 2021; **7**(5).
92. Zhao B, Liu C, Fan J, Ma A, He W, Hu Y, Zhao Y. Transmission and Drug Resistance Genotype of Multidrug-Resistant or Rifampicin-Resistant Mycobacterium tuberculosis in Chongqing, China. *Microbiol Spectr* 2022; **10**(5).

93. Fernández-Morales EA, Bermudez G, Montero H, Luzania-Valerio M, Zenteno-Cuevas R. Whole genome characterization, and geographical distribution of M. tuberculosis in central region of Veracruz, Mexico. *Braz J Infect Dis* 2022; **26**(3): 102357.
94. Yin J, Zhang H, Gao Z, et al. Transmission of multidrug-resistant tuberculosis in Beijing, China: An epidemiological and genomic analysis. *Front Public Health* 2022; **10**: 1019198.
95. Walter KS, Dos Santos PCP, Gonçalves TO, et al. The role of prisons in disseminating tuberculosis in Brazil: A genomic epidemiology study. *Lancet Reg Health Am* 2022; **9**.
96. López-Rocha E, Juárez-Álvarez J, Riego-Ruiz L, et al. Genetic diversity of the Mycobacterium tuberculosis complex in San Luis Potosí, México. *BMC Res Notes* 2013; **6**: 172.
97. Conceição EC, Rastogi N, Couvin D, et al. Genetic diversity of Mycobacterium tuberculosis from Pará, Brazil, reveals a higher frequency of ancestral strains than previously reported in South America. *Infect Genet Evol* 2017; **56**: 62-72.
98. Munro-Rojas D, Fernandez-Morales E, Zarrabal-Meza J, et al. Genetic diversity of drug and multidrug-resistant Mycobacterium tuberculosis circulating in Veracruz, Mexico. *PLoS One* 2018; **13**(3): e0193626.
99. Maguga-Phasha NTC, Munyai NS, Mashinya F, Makgatho ME, Mbajiorgu EF. Genetic diversity and distribution of Mycobacterium tuberculosis genotypes in Limpopo, South Africa. *BMC Infect Dis* 2017; **17**(1): 764.
100. Maharjan B, Nakajima C, Isoda N, et al. Genetic diversity and distribution dynamics of multidrug-resistant Mycobacterium tuberculosis isolates in Nepal. *Sci Rep* 2018; **8**(1): 16634.
101. Sanabria GE, Sequera G, Aguirre S, et al. Phylogeography and transmission of Mycobacterium tuberculosis spanning prisons and surrounding communities in Paraguay. *Nat Commun* 2023; **14**(1).
102. Affolabi D, Faihun F, Sanoussi N, et al. Possible Outbreak of Streptomycin-Resistant Mycobacterium tuberculosis Beijing in Benin. *EMERGING INFECTIOUS DISEASES* 2009; **15**(7): 1123-5.
103. Chen L, Pang Y, Ma L, et al. First Insight into the Molecular Epidemiology of Mycobacterium tuberculosis Isolates from the Minority Enclaves of Southwestern China. *Biomed Res Int* 2017; **2017**: 2505172.
104. Liu Z, Pang Y, Chen S, Wu B, He H, Pan A, Wang X. A First Insight into the Genetic Diversity and Drug Susceptibility Pattern of Mycobacterium tuberculosis Complex in Zhejiang, China. *Biomed Res Int* 2016; **2016**: 8937539.
105. France AM, Grant J, Kammerer JS, Navin TR. A field-validated approach using surveillance and genotyping data to estimate tuberculosis attributable to recent transmission in the United States. *Am J Epidemiol* 2015; **182**(9): 799-807.
106. Shea KM, Kammerer JS, Winston CA, Navin TR, Horsburgh CR, Jr. Estimated rate of reactivation of latent tuberculosis infection in the United States, overall and by population subgroup. *Am J Epidemiol* 2014; **179**(2): 216-25.
107. Xu AM, He CJ, Cheng X, et al. Distribution and identification of Mycobacterium tuberculosis lineage in Kashgar prefecture. *BMC Infect Dis* 2022; **22**(1): 312.
108. Li Y, Hu Y, Mansjö M, et al. The Epidemiological Significance and Temporal Stability of Mycobacterial Interspersed Repetitive Units-Variable Number of Tandem Repeats-Based Method Applied to Mycobacterium tuberculosis in China. *Int J Environ Res Public Health* 2018; **15**(4).

109. Tuite AR, Guthrie JL, Alexander DC, et al. Epidemiological evaluation of spatiotemporal and genotypic clustering of Mycobacterium tuberculosis in Ontario, Canada. *Int J Tuberc Lung Dis* 2013; **17**(10): 1322-7.
110. Huang CC, Trevisi L, Becerra MC, et al. Spatial scale of tuberculosis transmission in Lima, Peru. *Proc Natl Acad Sci U S A* 2022; **119**(45): e2207022119.
111. Al-Mahrouqi S, Ahmed R, Al-Azri S, et al. Dynamics of Mycobacterium tuberculosis Lineages in Oman, 2009 to 2018. *Pathogens* 2022; **11**(5).
112. Abadía E, Sequera M, Ortega D, et al. Mycobacterium tuberculosis ecology in Venezuela: epidemiologic correlates of common spoligotypes and a large clonal cluster defined by MIRU-VNTR-24. *BMC Infect Dis* 2009; **9**: 122.
113. Baker CR, Barilar I, de Araujo LS, et al. Use of High-Resolution Geospatial and Genomic Data to Characterize Recent Tuberculosis Transmission, Botswana. *Emerg Infect Dis* 2023; **29**(5): 977-87.
114. Lee SS, Chan DPC, Wong NS, Lui GCY, To KW, Kam JKM. Distribution of molecular strains of Mycobacterium tuberculosis in an intermediate burden Asia Pacific city. *Epidemiol Infect* 2021; **149**: e134.
115. Li M, Lu L, Jiang Q, et al. Genotypic and spatial analysis of transmission dynamics of tuberculosis in Shanghai, China: a 10-year prospective population-based surveillance study. *Lancet Reg Health West Pac* 2023; **38**: 100833.
116. Said H, Ratabane J, Erasmus L, et al. Distribution and Clonality of drug-resistant tuberculosis in South Africa. *BMC Microbiol* 2021; **21**(1): 157.
117. Baranov AA, Mariandyshev AO, Mannsåker T, Dahle UR, Bjune GA. Molecular epidemiology and drug resistance of widespread genotypes of Mycobacterium tuberculosis in northwestern Russia. *Int J Tuberc Lung Dis* 2009; **13**(10): 1288-93.
118. Renardy ME, Gillen C, Yang Z, Mukasa L, Bates J, Butler R, Kirschner DE. Disease phenotypic and geospatial features vary across genetic lineages for Tuberculosis within Arkansas, 2010-2020. *PLOS Glob Public Health* 2023; **3**(2): e0001580.
119. Rodríguez NA, Lirola MM, Chaves F, et al. Differences in the robustness of clusters involving the Mycobacterium tuberculosis strains most frequently isolated from immigrant cases in Madrid. *Clin Microbiol Infect* 2010; **16**(10): 1544-54.
120. Izumi K, Ohkado A, Uchimura K, et al. Detection of Tuberculosis Infection Hotspots Using Activity Spaces Based Spatial Approach in an Urban Tokyo, from 2003 to 2011. *PLoS One* 2015; **10**(9): e0138831.
121. Noppert GA, Yang Z, Clarke P, Davidson P, Ye W, Wilson ML. Contextualizing tuberculosis risk in time and space: comparing time-restricted genotypic case clusters and geospatial clusters to evaluate the relative contribution of recent transmission to incidence of TB using nine years of case data from Michigan, USA. *Ann Epidemiol* 2019; **40**: 21-7.e3.
122. Miyahara R, Piboonsiri P, Chiyasirinroje B, et al. Risk for Prison-to-Community Tuberculosis Transmission, Thailand, 2017-2020. *Emerg Infect Dis* 2023; **29**(3): 477-83.
123. Chen YY, Chang JR, Wu CD, et al. Combining molecular typing and spatial pattern analysis to identify areas of high tuberculosis transmission in a moderate-incidence county in Taiwan. *Sci Rep* 2017; **7**(1): 5394.
124. Xu G, Mao X, Wang J, Pan H. Clustering and recent transmission of Mycobacterium tuberculosis in a Chinese population. *Infect Drug Resist* 2018; **11**: 323-30.

125. Asare P, Otchere ID, Bedeley E, et al. Whole Genome Sequencing and Spatial Analysis Identifies Recent Tuberculosis Transmission Hotspots in Ghana. *Front Med (Lausanne)* 2020; **7**: 161.
126. Oostvogels S, Ley SD, Heupink TH, et al. Transmission, distribution and drug resistance-conferring mutations of extensively drug-resistant tuberculosis in the Western Cape Province, South Africa. *Microb Genom* 2022; **8**(4).
127. Huang SF, Su WJ, Dou HY, et al. Association of Mycobacterium tuberculosis genotypes and clinical and epidemiological features - a multi-center study in Taiwan. *Infect Genet Evol* 2012; **12**(1): 28-37.
128. Grandjean L, Iwamoto T, Lithgow A, et al. The Association between Mycobacterium Tuberculosis Genotype and Drug Resistance in Peru. *PLoS One* 2015; **10**(5): e0126271.
129. Ratovonirina NH, Rakotosamimanana N, Razafimahatratra SL, et al. Assessment of tuberculosis spatial hotspot areas in Antananarivo, Madagascar, by combining spatial analysis and genotyping. *BMC Infect Dis* 2017; **17**(1): 562.
130. Hu Y, Mathema B, Zhao Q, et al. Acquisition of second-line drug resistance and extensive drug resistance during recent transmission of Mycobacterium tuberculosis in rural China. *Clin Microbiol Infect* 2015; **21**(12): 1093.e9-.e18.
131. Verma A, Schwartzman K, Behr MA, Zwerling A, Allard R, Rochefort CM, Buckeridge DL. Accuracy of prospective space-time surveillance in detecting tuberculosis transmission. *Spat Spatiotemporal Epidemiol* 2014; **8**: 47-54.
132. Phyu AN, Aung ST, Palittapongarnpim P, et al. Genomic Sequencing Profiles of Mycobacterium tuberculosis in Mandalay Region, Myanmar. *Tropical Medicine and Infectious Disease* 2023; **8**(4): 239.
133. Li Y-f, Yang Y, Kong X-l, et al. Transmission dynamics and phylogeography of Mycobacterium tuberculosis in China based on whole-genome phylogenetic analysis. *International Journal of Infectious Diseases* 2024; **140**: 124-31.
134. Castro-Rodriguez B, León-Ordóñez K, Franco-Sotomayor G, et al. Population structure of Mycobacterium tuberculosis in El Oro: a first insight into Ecuador-Peru tuberculosis transmission. *Journal of Infection and Public Health* 2024; **17**(3): 527-34.
135. Utpatel C, Zavaleta M, Rojas-Bolivar D, et al. Prison as a driver of recent transmissions of multidrug-resistant tuberculosis in Callao, Peru: a cross-sectional study. *The Lancet Regional Health–Americas* 2024; **31**.
136. Ogwang MO, Diero L, Ng'ong'a F, Magoma G, Mutharia L, Imbuga M, Ngugi C. Strain structure analysis of Mycobacterium tuberculosis circulating among HIV negative, positive and drug resistant TB patients attending chest clinics in Western Kenya. *BMC Pulmonary Medicine* 2023; **23**(1): 497.
137. Yuen CM, Huang C-C, Millones AK, et al. Utility of Mycobacterium tuberculosis genome sequencing snapshots to assess transmission dynamics over time. *The Journal of Infectious Diseases* 2024; **229**(5): 1493-7.
138. Yang J, Lu Y, Chen Y, Wang Y, Wang K. Whole Genome Sequence-Based Analyses of Drug Resistance Characteristics, Genetic Diversity, and Transmission Dynamics of Drug-Resistant Mycobacterium tuberculosis in Urumqi City. *Infection and Drug Resistance* 2024: 1161-9.
139. Che Y, Li X, Chen T, et al. Transmission dynamics of drug-resistant tuberculosis in Ningbo, China: an epidemiological and genomic analysis. *Frontiers in Cellular and Infection Microbiology* 2024; **14**: 1327477.

140. Lan Y, Crudu V, Ciobanu N, et al. Identifying local foci of tuberculosis transmission in Moldova using a spatial multinomial logistic regression model. *EBioMedicine* 2024; **102**.
141. Liu Z, Li X, Xiong H, et al. Genomic and spatial analysis reveal the transmission dynamics of tuberculosis in areas with high incidence of Zhejiang, China: A prospective cohort study. *Infection, Genetics and Evolution* 2024; **121**: 105603.
142. Said H, Kachingwe E, Gardee Y, et al. Determining the risk-factors for molecular clustering of drug-resistant tuberculosis in South Africa. *BMC Public Health* 2023; **23**(1): 2329.

Preferred Reporting Items for Systematic reviews and Meta-Analyses extension for Scoping Reviews (PRISMA-ScR) Checklist

SECTION	ITEM	PRISMA-ScR CHECKLIST ITEM	REPORTED ON PAGE #
**TITLE**			
Title	1	Identify the report as a scoping review.	1
**ABSTRACT**			
Structured summary	2	Provide a structured summary that includes (as applicable): background, objectives, eligibility criteria, sources of evidence, charting methods, results, and conclusions that relate to the review questions and objectives.	1; unstructured as required but including all items.
**INTRODUCTION**			
Rationale	3	Describe the rationale for the review in the context of what is already known. Explain why the review questions/objectives lend themselves to a scoping review approach.	2
Objectives	4	Provide an explicit statement of the questions and objectives being addressed with reference to their key elements (e.g., population or participants, concepts, and context) or other relevant key elements used to conceptualize the review questions and/or objectives.	2
**METHODS**			
Protocol and registration	5	Indicate whether a review protocol exists; state if and where it can be accessed (e.g., a Web address); and if available, provide registration information, including the registration number.	2
Eligibility criteria	6	Specify characteristics of the sources of evidence used as eligibility criteria (e.g., years considered, language, and publication status), and provide a rationale.	2
Information sources*	7	Describe all information sources in the search (e.g., databases with dates of coverage and contact with authors to identify additional sources), as well as the date the most recent search was executed.	2
Search	8	Present the full electronic search strategy for at least 1 database, including any limits used, such that it could be repeated.	2

SECTION	ITEM	PRISMA-ScR CHECKLIST ITEM	REPORTED ON PAGE #
Selection of sources of evidence†	9	State the process for selecting sources of evidence (i.e., screening and eligibility) included in the scoping review.	2-3
Data charting process‡	10	Describe the methods of charting data from the included sources of evidence (e.g., calibrated forms or forms that have been tested by the team before their use, and whether data charting was done independently or in duplicate) and any processes for obtaining and confirming data from investigators.	3
Data items	11	List and define all variables for which data were sought and any assumptions and simplifications made.	3
Critical appraisal of individual sources of evidence§	12	If done, provide a rationale for conducting a critical appraisal of included sources of evidence; describe the methods used and how this information was used in any data synthesis (if appropriate).	Not applicable
Synthesis of results	13	Describe the methods of handling and summarizing the data that were charted.	2-3
**RESULTS**			
Selection of sources of evidence	14	Give numbers of sources of evidence screened, assessed for eligibility, and included in the review, with reasons for exclusions at each stage, ideally using a flow diagram.	3-4
Characteristics of sources of evidence	15	For each source of evidence, present characteristics for which data were charted and provide the citations.	4-5
Critical appraisal within sources of evidence	16	If done, present data on critical appraisal of included sources of evidence (see item 12).	Not applicable
Results of individual sources of evidence	17	For each included source of evidence, present the relevant data that were charted that relate to the review questions and objectives.	3-6
Synthesis of results	18	Summarize and/or present the charting results as they relate to the review questions and objectives.	6-11
**DISCUSSION**			
Summary of evidence	19	Summarize the main results (including an overview of concepts, themes, and types of evidence available), link to the review questions and objectives, and consider the relevance to key groups.	11-12
Limitations	20	Discuss the limitations of the scoping review process.	12-13
Conclusions	21	Provide a general interpretation of the results with respect to the review questions and objectives, as well as potential implications and/or next steps.	13-14
**FUNDING**			
Funding	22	Describe sources of funding for the included sources of evidence, as well as sources of funding for the scoping review. Describe the role of the funders of the scoping review.	14

JBI = Joanna Briggs Institute; PRISMA-ScR = Preferred Reporting Items for Systematic reviews and Meta-Analyses extension for Scoping Reviews.
* Where *sources of evidence* (see second footnote) are compiled from, such as bibliographic databases, social media platforms, and Web sites.

† A more inclusive/heterogeneous term used to account for the different types of evidence or data sources (e.g., quantitative and/or qualitative research, expert opinion, and policy documents) that may be eligible in a scoping review as opposed to only studies. This is not to be confused with *information sources* (see first footnote).
‡ The frameworks by Arksey and O'Malley (6) and Levac and colleagues (7) and the JBI guidance (4, 5) refer to the process of data extraction in a scoping review as data charting.
§ The process of systematically examining research evidence to assess its validity, results, and relevance before using it to inform a decision. This term is used for items 12 and 19 instead of "risk of bias" (which is more applicable to systematic reviews of interventions) to include and acknowledge the various sources of evidence that may be used in a scoping review (e.g., quantitative and/or qualitative research, expert opinion, and policy document).

From: Tricco AC, Lillie E, Zarin W, O'Brien KK, Colquhoun H, Levac D, et al. PRISMA Extension for Scoping Reviews (PRISMAScR): Checklist and Explanation. Ann Intern Med. 2018;169:467–473. doi: 10.7326/M18-0850.
